# Patients’ experiences and usability of a self-directed m-health exercise intervention for knee osteoarthritis: a qualitative study

**DOI:** 10.1136/bmjopen-2025-100608

**Published:** 2025-06-16

**Authors:** Valerie Dieter, Joana Schmidt, Inga Krauss

**Affiliations:** 1 Department of Sports Medicine, Medical Clinic, University Hospital of Tübingen, Tübingen, Germany; 2 Interfaculty Research Institute for Sports and Physical Activity, Tübingen, Germany

**Keywords:** Exercise, Digital Technology, eHealth, Musculoskeletal disorders, Knee, QUALITATIVE RESEARCH

## Abstract

**Objectives:**

The aim of the study was to analyse patients’ experiences, usability and acceptability of a 12-week self-directed m-health exercise intervention for patients with knee osteoarthritis (OA).

**Design and participants:**

This qualitative study was included in a randomised controlled trial. After completion of a 12-week m-health exercise intervention, 14 structured telephone interviews were conducted in patients with knee OA. The interviews were audio recorded, transcribed and analysed using a combined deductive and inductive content analysis approach.

**Setting:**

The study was conducted at a university hospital in southern Germany.

**Results:**

Four themes were identified: (1) facilitators and (2) barriers for (correct) digital exercise participation, (3) ease of use and (4) outcomes. Participants valued ease of use, comprehensibility, flexibility and certain app features (eg, push notifications as reminders, instruction videos, real-time biofeedback) that facilitated guidance, control and motivation to complete exercises. However, some participants requested some kind of human support.

**Conclusions:**

The participants demonstrated predominantly positive experiences and acceptability with the self-directed m-health exercise intervention. As some participants lacked human support, we suggest that the optimal treatment option should be selected according to the preferences and needs of the individual patient.

**Trial registration number:**

DRKS00023269.

**STRENGTHS AND LIMITATIONS OF THIS STUDY**

- The interviews were conducted within a time window of 2–5 weeks following intervention completion, enabling adequate recall of the mobile app and the exercise programme.
- The independent development of themes and subthemes by two researchers, and the subsequent review and discussion process with a third researcher, led to rigorous results.
- No purposive sampling (eg, with age, sex, technical affinity, positive or negative outcomes) was included.

## Introduction

 Osteoarthritis (OA) is a highly prevalent, degenerative joint disease that affects almost 18% of German adults.^1^ The burden of OA increases with age, female sex and higher body mass index. The knee joint is most commonly affected.^2^ In several clinical guidelines, exercise and education are recommended as first-line treatments for knee OA.^3^ However, less than 50% of the German OA population received physical therapy at least once in the previous 12 months. In particular, men and individuals with a low household income were found to use physical therapy less frequently.^4^ Overall, the recommendation for first-line non-pharmacological therapeutic exercise treatment is even less than 40%.^5^ This is the case even though the beneficial effects of exercise are comparable to those of analgesics and oral non-steroidal anti-inflammatory drugs, and exercise therapy is known to have few side effects.^6 7^ In order to address the current deficiencies in healthcare, the utilisation of digital health technologies in the delivery of exercise programmes could be increased in the future. It can be assumed that this could additionally facilitate patients’ access and adherence to unsupervised home-based exercise. By definition, digital health technologies integrate information and communication tools that can be used to improve accessibility and efficiency of healthcare.^8 9^ In this context, digital health is an umbrella term that encompasses electronic health (e-health), mobile health (m-health) and telehealth,^9^ whereby m-health technologies specifically focus on mobile communication tools.^10^ Overall, people with knee OA have already demonstrated positive experiences when using an e-Health exercise intervention. They valued the technical ease of use, simplicity and comprehensiveness of the information provided, regular prompts to support participation in self-directed exercise programmes and a credible content source (eg, universities).^8^ Additional features, including feedback mechanisms, exercise instructions, exercise images and videos, can further support correct exercise conduction without supervision^11^ and thus constitute favourable determinants for the implementation of the intervention. Conversely, the absence of human presence could be perceived as a barrier by at least some patients.^11 12^ The potential uncertainty regarding correct exercise performance is a reason for stopping training and can ultimately lead to non-adherence.^13^ In this context, the special group of sensor-assisted digital exercise delivery represents a promising avenue for enhancing the support available to patients exercising at home. These technologies can provide additional guidance on the correct exercise execution (eg, verbal, visual and auditory) and appropriate training load, using real-time biofeedback. Consequently, they have the potential to replace supervision to a certain extent.

To date, most qualitative studies on patients with knee OA have investigated the feasibility and acceptability of web-based programmes^11 12^ or supervised digital delivery modes (eg, Skype).^14^ Consequently, there remains a limited understanding of patients’ experiences, usability and acceptability when using a self-directed app-delivered and sensor-assisted exercise intervention for patients with knee OA. Ultimately, addressing this gap is crucial for the successful implementation of a programme.^15^ In this context, existing studies on digital exercise interventions have largely overlooked the distinction between different types of digital health technologies. However, this is a critical oversight, as varying levels of technological support may present unique challenges for patients, potentially influencing their engagement and outcomes.

To investigate this, a randomised controlled pilot trial (RCT) was conducted involving a 12-week self-directed m-Health exercise intervention (re.flex). The efficacy of the intervention on knee pain and further patient-reported outcome measures was evaluated in 61 participants and published elsewhere.^16^ This qualitative examination aimed to identify patients’ perspectives on facilitators and barriers, usability and acceptability of a self-directed app-delivered and sensor-delivered exercise intervention. In this context, ‘acceptability’ was defined as a construct that reflects the evaluation of the m-health exercise app as a suitable method for delivering an exercise intervention, based on patients’ experiences and needs.

## Methods

### Design

The qualitative study design was integrated within an RCT (German clinical trial register: DRKS00023269)^16^ (for the study protocol, see online supplemental appendix 3). The study is reported following the Consolidated criteria for Reporting Qualitative research checklist.^17^

### Participants

Participants were a subsample of those allocated to the control arm of the pilot RCT, who had completed their 12-week intervention after the initial 12-week control period. Initial recruitment for the RCT was obtained via advertisements in regional newspapers and mailing lists. Eligibility criteria included a diagnosis of knee OA with unicondylar tibiofemoral complaints and at least a Knee Osteoarthritis Outcome Score (0–100, worst to best) of the subscale pain ≤60 points at the time of screening. In addition, participants were required to have access to a tablet or mobile phone with an iOS operating system. Details on full RCT eligibility criteria are reported elsewhere.^16^ Participants were randomly selected. The sample size was specified a priori according to previous qualitative research studies examining digital exercise interventions for patients with knee OA.^11 12^ No purposive sampling was included. After selection, the potential participants were contacted by telephone and asked for their consent to participate in the interview survey. Participation was based on voluntary consent and was independent of the previous RCT study. In case of participation, an extended consent form was signed.

### Intervention

**Figure 1:**
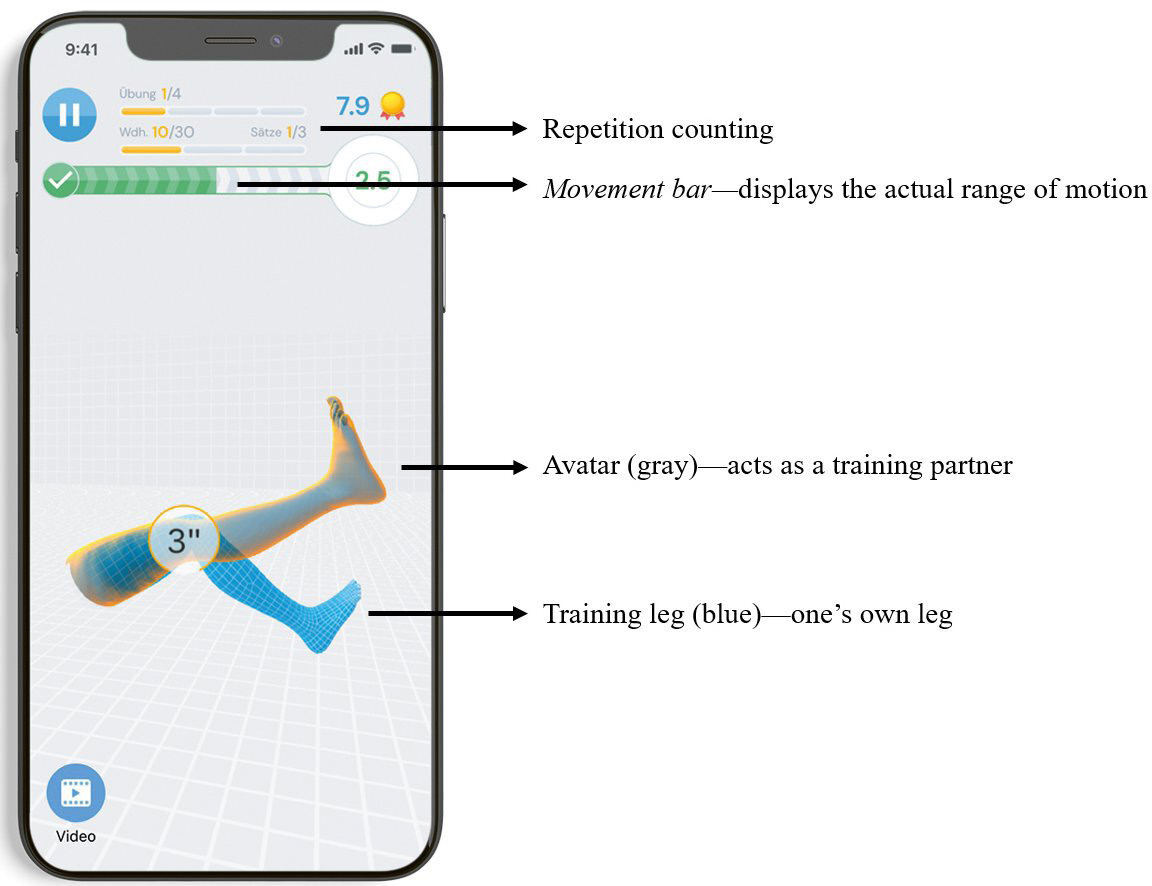
Screenshot with features of the re.flex app. This screenshot illustrates the key features of the re.flex app. In the upper section, repetition counting and a movement bar displaying the actual range of motion of the user are provided. The grey avatar leg acts as a training partner, demonstrating the target condition. The blue training leg represents the patient’s virtual limb. The goal is to align both avatars in order to execute exercises with the correct technique, range of motion and movement velocity.

Details of the digital app-based exercise intervention conducted by the participants prior to the interview are described elsewhere.^16^ The intervention included a 12-week self-directed home exercise programme with three sessions per week. Exercises were guided and monitored by use of the training app *re.flex* (figure 1) and two motion sensors that were attached proximally and distally to the affected knee joint. Thereby, the app provided exercise videos and exercise instructions. The utilisation of motion sensors enabled the visualisation of the patient’s virtual limb in conjunction with an avatar reflecting the target condition. The goal was to align both avatars. Biofeedback allowed control over movement execution, predefined range of motion, movement velocity and repetition counting. Visual and auditive feedback were provided by a movement bar, a real-time rating of movement quality and an auditive signal whenever reaching the end position of a movement. Verbal instructions were given if an exercise was not performed correctly. Further app features allowed push notifications to remind for upcoming training sessions, rating of perceived pain and exertion, to pause or skip exercises and to monitor training progress with statistics. The programme comprised exercises for strengthening, balance, mobilisation and stretching with a special focus on musculature of knee extensors, knee flexors and hip abductors. The exercises were predefined for each session but could be individually tailored by choosing between two different intensity levels or varying the resistance of the bands. In case of technical and medical issues, participants could contact the provider using the app messenger service. In the context of the study, this function was supervised by the study personnel. The content of the exercise programme was developed by researchers and physical therapists (eg, VD, IK) from the Department of Sports Medicine at the University Hospital of Tübingen and specifically designed for knee OA patients. The selection of exercises was based on disease-specific recommendations^18^,^20^ and experiences of previous clinical trials in which similar exercise interventions for patients with hip and knee OA were employed.^21^,^24^

### Interviews

Structured telephone interviews were conducted by two study staff members (IS and AM, student assistants during Health Management Studies/Sports Science Studies) who were not involved in intervention design, the recruitment for and the evaluation of the RCT. The interview guide was developed by the authors VD (sports scientist, RCT coordinator) and IK (sports scientist and physiotherapist) and addressed patients’ (positive and negative) experiences with the app and its features during the course of the exercise intervention. In addition, the patients’ needs and perspectives on usability, the design of the app, and suggestions for improvement to optimise app functionality were gathered (online supplemental appendix 1). Interviewers had no previous experience in guided interviews but were introduced and trained by the RCT coordinator. The participants had met one of the interviewers during the RCT assessment appointments. Interviews were audio recorded and transcribed verbatim by AM and JS. Audio records and transcripts have been pseudonymised and stored with restricted access to comply with data protection guidelines. Transcripts were not returned to participants for comment or correction, nor could participants provide feedback on the findings. No data saturation was discussed and no repeated interviews were conducted.

### Data analysis

We used MAXQDA V.24 (VERBI, Berlin, Germany) for organising the pseudonymised transcripts. Data were analysed following the steps according to qualitative content analysis by Kuckartz and Rädiker.^25^ A first category system was developed by one researcher (JS), combining a deductive (derivation of content of the interview guide) and inductive (elaboration of additional themes) approach. JS was not involved in interview data collection or design and evaluation of the RCT. The category system was discussed by all authors while creating new themes. In the next step, two researchers (JS and VD) independently grouped codes of all interviews into themes and subthemes. They compared and discussed results in order to seek agreement. A third researcher (IK) was included to refine subthemes and themes names and definitions. The analysis process was iterative and reflective with forward and backward movement within the different steps. After completing the analysis with data reduction, JS and VD translated all quotes from German into English language.

### Patient and public involvement

None.

## Results

### Participant characteristics

Out of 16 randomly selected participants, 14 gave consent and were interviewed between 6 May 2021 and 19 May 2021. All participants completed the interview within a time window of 2–5 weeks after intervention completion. Two other participants declined to participate—one for time reasons in combination with a rehabilitation stay and one due to lack of interest. The average duration of the interviews was 57 min. Table 1 reports the baseline characteristics of the interviewed participants.

**Table 1:** Study population characteristics

	n=14
Gender (♂ / ♀), n (%)	7 (50)/7 (50)
Age in years, mean (SD)	63.9 (6.9)
Body mass index in kg/m², mean (SD)	28.0 (4.5)
Education, n (%)	
Academic education	10 (71)
Vocational education	4 (29)
Employment, n (%)	
Employed	7 (50)
Retired	7 (50)
Previous experience with exercise therapy, n (%)	
Very large	0 (0)
Large	2 (14)
Moderate	6 (43)
Low	6 (43)
Very low	0 (0)
Previous participation in a hip/knee sports group, n (%)	
Yes	0 (0)
No	14 (100)
Technical affinity*, mean (SD)	2.7 (0.6)
Baseline knee pain†, mean (SD)	57.7 (18.2)

* 5-point Likert scale from 1 (not true at all) to 5 (fully true).

† Self-reported knee pain in the past week rated with the subscale pain of the Knee Osteoarthritis Outcome Score, 0–100, worse to best.

### Content analysis

Four main themes were identified during the data analysis. Additional details on themes, subthemes and supporting quotes can also be found in online supplemental appendix 2.

#### Theme 1: facilitators for (correct) digital exercise participation

##### App features for guidance, support and monitoring of correct exercise execution

An instruction of the exercises with a combination of videos from multiple perspectives and text descriptions was found to be useful by participants because of its ability to show even the smallest details. Additionally, an avatar acting as a training partner to demonstrate and control the exercises, auditory and visual signals when the preset target movement range was reached and verbal feedback were also rated as helpful and supportive, particularly insofar as they serve to mitigate uncertainty to conduct exercises without supervision, provide a control mechanism and offer real-time feedback on correct or incorrect movement execution.

Yes, that [avatar acting as a training partner] helped me a lot with some exercises that I didn't understand straight away […], this highlighted color [avatar leg] and your own [patient’s virtual limb], you could see, I have to stretch it [the leg] differently or I don't have to hold it that low […] that was simply a good support. [AT51]

Yes, that [verbal feedback] was actually almost always helpful, for example when you were told ‘stretch your leg a bit more’ or ‘your knee is too high’ […], I would say that was true in most cases, it was then comprehensible, correctable, and you could also synchronize it to some extent with the avatar. [AT58]

##### Flexibility

The flexibility to conduct exercises at any time and in any location was seen as a key benefit of the app. The utilisation of m-health for the delivery of exercises was perceived as an optimal alternative for training at home, particularly for individuals with limited time availability or access to other training options.

I think another big advantage is that you can simply integrate it into your day, that you are flexible, you don’t have to be somewhere at a certain time and you don’t lose any time […] getting from A to B to see a physiotherapist, you can just do it yourself at home. [AT38]

##### App features to increase motivation and regularity of the exercise programme

Participants emphasised that some of the app features, including the motion sensors that provide a control mechanism and objective monitoring of the frequency with which exercises were performed, the positive verbal feedback that was perceived as acknowledgement, the exercise overview and the reminder feature for the scheduled training days and times, were effective motivators for regular training. These features contribute to a sense of safety, control and enjoyment, which in turn increased the user’s motivation to adhere to the exercise programme.

I always felt so supported and guided, […]. I realized for myself that if I would not have had the reminder [push-notifications at training days] and the knowledge about the training days, I probably wouldn't have been so disciplined. [AT38]

##### Positive experiences with the exercise programme

Positive experiences with the exercise programme were linked to noticeable progress with recurring exercises and increased difficulty level, enjoyment while exercising, alternation of demanding strength exercises and relaxed mobilisation or stretching exercises, and a manageable training volume that could be easily integrated into everyday life.

### Theme 2: barriers for (correct) digital exercise participation

#### No on-site instruction by a health professional

Participants felt that in certain cases, for example, if pain occurred during exercise or if uncertainty was experienced during a presumably incorrect exercise execution, a health professional such as a physical therapist could have provided additional or more precise instructions or could have facilitated a more rapid correction of incorrect movements. In addition, the focus of a health professional would not have been limited to the knee; it could have also been extended to the entire body.

I sometimes need someone to say ‘your posture isn't quite okay or try this’. You do like to make evasive movements if it is painful somewhere […], so you want someone to tell you ’not like that‘. […] a trained professional who looks at you and says ’now do the exercise like this‘. That you simply have real control. Of course, you have control through the sensors and the app, but you can still cheat a bit. [AT10]

#### Technical issues

Due to technical issues, some participants encountered difficulties in conducting certain exercises. In certain instances, the calibration process required multiple attempts to accurately reproduce the correct position of the virtual training leg within the environment. A further issue was experienced with the sensor registration of performed exercise repetitions, which failed in some cases due to unreachable range of motion. This particularly concerned exercises involving resistance bands. The participants reported that technical issues had a detrimental effect on their motivation and training performance, as the affected exercise could not be completed.

Always assuming that the thing [app with the technical features] really works. Then that would be a really great thing for me [pause]. However, if these technical issues occur and I can't solve them, then that’s rather demotivating. [AT63]

#### Difficulties with the exercise programme

The participants identified barriers that were related to the exercise programme itself. One difficulty comprised individual exercises requiring high knee flexion in combination with high load on the affected leg (eg, one-sided exercises, wall squat) with the consequence that participants could have experienced pain. This finding seemed to be particularly pronounced among participants with a high body mass index.

#### Associated problems with app features

Not every incorrect exercise execution could be detected and admonished by the app. Participants reported accepting incorrect exercise execution in order to cheat the app in the absence of repetition counting due to failed sensor registration. In doing so, they sometimes did more repetitions than specified in order to register all repetitions as completed in the app. Moreover, if movements were repeatedly performed incorrectly, the verbal feedback with the error message was repeated again and again. This was sometimes perceived as annoying. Additionally, as only one leg was equipped with sensors, participants reported that the training with the non-sensor leg was done less accurately, faster and more carelessly.

### Theme 3: ease of use

#### Usability, feasibility and comprehensibility

Most participants found the app easy to use, the navigation easy to follow and the practical implementation and information easy to understand and self-explanatory. It was reported that the technical procedures before and during the training sessions had become routine.

I really didn’t have had any difficulties, the handling is okay, it is clear, understandable, self-explanatory, and it’s good. [AT10]

#### Handling and screen size

Several participants reported that the screen size of a smartphone was insufficient to view all available information and details, suggesting that a tablet would be a more suitable option. When exercising in the supine position, for some participants, it was a challenge to find an optimal position for the device in order to have a clear view and still be able to operate the app.

### Theme 4: outcomes

#### Positive outcomes

Most of the participants reported that they had benefited in some way from the exercise intervention. Many of them felt less pain, required less pain medication, noticed an improvement in function or were able to do everyday activities (eg, walking, cycling, climbing and descending stairs) for a longer time and with less discomfort. Several participants also experienced a noticeable increase in muscle strength and stability around the knee, as well as improved self-efficacy.

[…] but the pain has now decreased considerably thanks to all the exercises, I am taking far fewer painkillers and when I go in with pain, it hasn't got any worse. [AT56]

Well, I just wished or hoped that I would build up muscle in my knee and thus, become more pain-free, and that has actually turned out to be the case [pause]. So, it improved, I can walk the stairs much better, I can walk better at all. […] Overall, I was very happy with the app and also with the results. [AT53]

#### Not met outcome expectations

A few participants reported that their expectations of participating in the exercise study were not met, as they were unable to make any progress in terms of muscle building or pain relief. As a result, their motivation for participating in the study and weekly exercise sessions, which was to delay or avoid knee surgery, may not have been fulfilled. Moreover, one participant stated that the absence of training success resulted in a loss of enjoyment in continuing the exercises.

As I said, the expectation that it will really improve to such an extent that surgery is no longer an option is not given, so that I will probably expect one in the next six or nine months. I didn't really have had any other expectations. [AT58]

## Discussion

### Principal findings

This study investigated experiences, usability and acceptability of a 12-week self-directed m-health exercise intervention for patients with knee OA. Most participants reported positive outcomes, including reduced pain, an improvement in function and daily activities and an increase in muscle strength. Overall, the participants valued the ease of use, comprehensibility and self-explanatory nature of the m-health-supported exercise programme. The flexibility to conduct exercises at any time and in any location was seen as a key benefit. In addition, app features including videos, real-time biofeedback, verbal feedback and the reminder for upcoming exercise sessions via push notifications were found to be useful, supportive and motivating in order to guide and control regular exercising. However, some participants would have found some kind of human supervision in combination with the m-health intervention even more beneficial. In general, technical issues had a detrimental effect on motivation and adherence to exercise.

The participants in our study identified the ease of use, comprehensibility and self-explanatory nature of the m-health-supported exercise programme as being of significant importance. This is in accordance with findings from other qualitative studies examining experiences with self-directed or (partly) supervised e-health exercise interventions in patients with hip or knee OA. They also emphasised the importance of simplicity and comprehensibility in the use of e-health technologies and highlighted their potential impact on adherence and motivation.^11 13 26^ Furthermore, push notifications as a reminder for regular exercise were valued by the participants. In other qualitative studies, email reminders and SMS messages were used in order to prompt or push participants to perform exercises.^11 27^ Most participants found the reminders to be a positive facilitator and motivator.^27^ Other app features that were described as facilitators and motivators for exercise participation were instructional videos,^27^ the positive effect of monitoring when they knew someone was watching^13^ and a feedback system.^27^ These findings are consistent with the results of our study, in which participants identified videos, real-time biofeedback, verbal feedback and push notifications as supportive and motivating tools for regular exercise participation.

Flexibility to undertake exercises at any time and in any location was found to be a key benefit of the m-health exercise delivery in the current study. In particular, it was considered to be a highly appreciated alternative for training at home, which could be conducted without wasting time travelling to another location. This finding was corroborated by two further studies using e-health technologies to promote physical activity, exercise and education, either without human support or asynchronous chat support.^12 27^ Overall, the combination of flexibility with appropriate exercise guidance using e-health technologies could facilitate greater access to conservative OA exercise treatment for a larger number of individuals. This also has a societal value, as exercise is classified as a first-line treatment in knee and hip OA care.^3^

However, despite the numerous benefits and facilitating factors associated with participating in m-health delivered exercise, the participants identified a significant barrier in the absence of human supervision and on-site instruction by a health professional. The participants indicated a lack of additional or more precise instructions, particularly if pain occurred during the exercise or if the exercises were performed incorrectly. Studies investigating self-directed e-health interventions have reported similar findings.^11 12^ Participants suggested that e-health interventions could be improved by the provision of human support when needed, for example, in the context of clinical questions.^11^ This appears to be of particular importance in patients with comorbidities,^12^ who may require more tailored exercise programmes. In studies incorporating asynchronous chat support from a physical therapist or a blended intervention of e-health exercise, participants valued the daily contact compared with traditional care^27^ and perceived the support to be important and motivating to continue exercising.^26 27^ The ability of physical therapists to tailor exercise programmes according to patients’ needs was described as a key benefit in a blended care intervention.^26^ It appears that individuals seek support from a health professional, at least on a basic level, enabling that someone monitors their progress, guides progression and provides motivation and feedback.^28^ In addition, the incorporation of human interaction with a health professional via telehealth or blended care in digital exercise delivery may address the importance of adequately preparing patients for upcoming digital exercise sessions by empowering them for correct and safe exercise technique, answering questions, providing reassurance or managing technological issues.^14 29^ To summarise, some kind of human support could be useful even in e-health exercise delivery, but the necessary amount of support is dependent on patients’ preferences and needs.

### Strengths and limitations

One strength of our study includes that all participants completed the interview within a time window of 2–5 weeks after intervention completion. This enabled adequate recall of the mobile app and the exercise programme. A potential bias associated with the researcher (JS) who developed the coding system could be mitigated, as she did not interact with the participants nor was she involved in the design or conduction of the study. Using an investigator triangulation approach, themes and subthemes were independently developed by two researchers (JS and VD), and subsequently reviewed and discussed by a third researcher (IK). Furthermore, the utilisation of audio recording, the aforementioned triangulation, and reflexivity strategies (eg, documentation sheets for the analysis process) in the research design served to strengthen the reliability of the findings. However, there were some limitations. First, the interviews were conducted by telephone, which may have resulted in less in-depth information of the interviews and more superficial responses due to the absence of personal interaction. Furthermore, the sample size was determined a priori. However, despite this limitation, inductive thematic saturation, as well as a priori thematic saturation, could be confirmed in the data set.^30^ Moreover, there was a potential selection bias, as no purposive sampling (eg, with age, sex, technical affinity, positive/negative outcomes) was included. This might have implications for the generalisability of the findings, as certain patient characteristics could be under-represented in the study sample. In addition, the participants self-selected to participate in the related RCT. This suggests that they had already developed an interest in attending an exercise programme with digital guidance and may, therefore, have had a more positive attitude on the use of digital technologies in exercise delivery. Lastly, the involvement of the researchers (VD and IK) in the development of the exercise programme and the conduction of the RCT could have affected findings.

### Conclusion and implications for clinical practice

Participants demonstrated positive experiences and acceptability of the self-directed m-health exercise intervention for knee OA. The majority of participants reported positive outcomes and valued ease of use, comprehensibility, flexibility and certain app features (eg, push notifications as reminders, instruction videos, real-time biofeedback) to facilitate guidance, control and motivation of the self-directed digital exercise participation. Nevertheless, some participants requested some kind of human support in combination with the digital intervention. For clinical practice, this study demonstrates that self-directed digital exercise interventions are not a ‘one-size-fits-all’ solution. Instead, potential advantages and disadvantages should be discussed with the individual patient, as personal and environmental context factors (eg, level of support, previous experience in physical exercise, technical affinity, access to healthcare providers) need to be considered when selecting the appropriate treatment option. In the future, further modifications, for example, the implementation of an automated feedback system reacting on exertion and pain levels from previous exercise sessions, could lead to a more individualised and tailored exercise intervention. Moreover, future research should focus on the comparison between supervised and self-directed digital exercise delivery in knee OA. In this context, it is crucial to identify the responder characteristics for the different delivery modes, thus enabling the recommendation of a best-fit solution for each patient, tailored to their individual characteristics.

## Supplementary material

10.1136/bmjopen-2025-100608 online supplemental file 1

10.1136/bmjopen-2025-100608 online supplemental file 2

10.1136/bmjopen-2025-100608 online supplemental file 3

## Data Availability

Data are available on reasonable request.
