# Supplementary material for: Patients’ experiences and usability of a self-directed m-health exercise intervention for knee osteoarthritis: a qualitative study
Source: BMJ Open. 2025 Jun 16;15(6):e100608. doi: 10.1136/bmjopen-2025-100608 (PMC12314823; doi:10.1136/bmjopen-2025-100608)
Supplement: online supplemental file 1 [file bmjopen-15-6-s001.docx]

# Appendix 1: Interview guide

Interviewleitfaden

Studie App-gestützte Trainingsintervention (re.flex)

# Forschungsfrage

Wie wurde die Interaktion mit der App erlebt und bewertet?

Wie sind die Usability und das Design der re.flex-App zu bewerten?

Welche Hinweise zur Optimierung lassen sich hieraus erarbeiten?

# Informationen zum Interview

Danke, dass Sie sich die nächsten circa 45-60 Minuten Zeit nehmen, um Ihre bisherigen Erfahrungen während der Studie „App-gestützte Trainingsintervention“ zu teilen. Die beantworteten Fragen dienen einem wissenschaftlichen Zweck und werden für die Weiterentwicklung und Optimierung der App genutzt.

Da wir Ihre Aussagen gerne auswerten und für diesen Zweck nutzen möchten, würden wir das Gespräch gerne aufzeichnen. Sind Sie damit einverstanden, dass wir das Interview für diesen Zweck aufnehmen?

Alle Audiodateien werden pseudonymisiert gekennzeichnet und nicht an Dritte weitergegeben. Die Aufnahmen werden nach dem Interview von dem Aufnahmegerät gelöscht und in einen Ordner geladen, auf den nur Studienbetreuer Zugriff haben.

Das Beenden des Interviews ist zu jeder Zeit und ohne Begründung möglich.

[AUFNAHME STARTEN]

Uns interessiert vor allem wie Sie die Interaktion mit der App erlebt sowie Benutzerfreundlichkeit und Design der App wahrgenommen haben. Zudem möchten wir erfahren, ob sich im Rahmen des Trainings mit der App Schwierigkeiten oder Sicherheitsbedenken aufgetan haben oder Sie Verbesserungsvorschläge zur Optimierung der Durchführbarkeit des app-gestützten Trainings haben.

Gibt es an dieser Stelle von Ihnen noch Fragen? Ansonsten möchte ich Sie noch bitten die Fragen offen und ehrlich zu beantworten.

# Einstieg

Freude und Abneigung

„Ice breaker“

1. Haben Sie das erste Mal mit einer App trainiert?
2. Wie haben Sie sich gefühlt (beim Nutzen/Trainieren mit der App)?
3. Welche Erfahrungen konnten Sie bisher während der Studie beim Training mit der App sammeln?

Formalia

1. Mit welchem Endgerät haben Sie das Training durchgeführt (Bei Nachfrage: Hinweis auf Smartphone, Tablet, ggf. Größe, wenn bekannt).
2. Wie lange nutzen Sie das Endgerät oder ein vergleichbares Vorgängermodell schon?

Allgemein

1. Welche Vorteile sehen Sie im Nutzen einer solchen App?
2. Was hat Ihnen an der App gefallen?
3. Welche Nachteile sehen Sie im Nutzen einer solchen App?
4. Was hat Ihnen an der App nicht gefallen?
5. Welche Schwierigkeiten hatten Sie beim Nutzen der App?

Layout und Darstellung/Design

1. War die Farbauswahl und Größe der Schaltflächen (Buttons) ausreichend an Ihre Bedürfnisse angepasst?
   1. War die Schrift gut zu lesen (beispielsweise in Hinblick auf Größe der Schrift, Schrifttyp oder sonstiges)?
2. Waren alle Schaltflächen (Buttons) gut ersichtlich?
3. Wussten Sie immer, für welchen Zweck diese eingesetzt werden sollten?
4. Ist die Menge an Informationen, die zeitgleich auf dem Bildschirm präsentiert werden, angemessen? (Stichwort: Übersichtlichkeit?)
5. Wie bewerten Sie die Wahl der Sprache, in der die Anweisungen in der App gestaltet sind? (Stichwort: leichte und verständliche Sprache)
6. Was fällt Ihnen positiv an dem Design der App auf?
7. Was würden Sie am Design der App gerne verbessern?
8. Können Sie uns bitte hierzu mitteilen, ob Sie Einschränkungen der Sehfähigkeit haben, die Sie bei der Nutzung der App beeinträchtigt haben (ggf. kommentieren)
9. Können Sie uns bitte hierzu mitteilen, ob Sie Einschränkungen der Hörfähigkeit haben, die Sie bei der Nutzung der App beeinträchtigt haben (ggf. kommentieren)

Technische Aspekte

Umgang mit den technischen Instrumenten (App und Orthese)

1. Wie empfanden Sie die Handhabung mit den Sensoren vor und während des Trainings (Gruppe A und AB)?
   1. Sensoren laden
   2. Sensoren/Orthese anlegen
   3. Sensoren verbinden
   4. Sensoren kalibrieren
   5. Handhabung während des Training
2. Wie empfanden Sie die Handhabung der Orthese vor und während des Trainings (nur Gruppe AB)?
   1. Orthese anlegen
   2. Handhabung während des Training
3. Wie empfanden Sie den Vorbereitungsprozess mit der technischen Ausrüstung im Vorfeld des Trainings?

Trainingsdurchführung mit der App

Anleitung des Trainings

1. Wie empfanden Sie die Anleitung des Trainings durch die App?
   1. Wie bewerten Sie die Video- und Übungsbeschreibung?
      1. Wie verständlich waren für Sie die Instruktionen der Übungen 1.) durch die Videos und II.) durch die schriftlichen Beschreibungen?
      2. Wie beurteilen Sie die gewählten Bildausschnitte und Perspektiven der Videos?
      3. Was hat Ihnen an den Instruktionsvideos/ -beschreibungen gefehlt?
   2. Wie empfanden Sie die Übungsdurchführung gemeinsam mit dem Avatar (das graue Vormachbein)?
   3. Wie empfanden Sie insgesamt die Handhabung eines Smartphones/Tablets während des Trainings?

Fehlfunktionen während der Nutzung

1. Haben Sie irgendwelche „Fehler/Bugs“ in Ihrer App gefunden?
   1. Wenn die App nicht reagierte oder abstürzte war es leicht sie neu zu starten und wieder in Gang zu bringen?
   2. Mussten Sie jemals eine Trainingseinheit aufgrund von technischen Schwierigkeiten abbrechen?
   3. Mussten Sie sich jemals aufgrund technischer Schwierigkeiten an den Support wenden? Wenn ja, waren die Informationen hilfreich um Ihre Probleme zu lösen oder hätten Sie sich mehr Hilfestellung gewünscht (wenn mehr Hilfestellung, welcher Art könnte/sollte diese aussehen?)

Allgemeine Menüführung

1. Bitte bewerten Sie die Verständlichkeit der Menüführung während der Trainingsdurchführung.
   1. Haben Sie bei der Bedienung der App etwas vermisst?
2. Welche Schwierigkeiten sind während des Trainings beim Navigieren durch die App aufgetreten?

Bewertung einzelner Menüpunkte/Features/Funktionen

1. Wie bewerten Sie die folgenden Menüpunkte/Funktionen?
   1. Balken mit Blase am Ende zur Orientierung der Bewegungsweite
   2. Verbales Feedback bei fehlerhafter Übungsdurchführung
   3. Avatar + Ihr dargestelltes Trainingsbein zur Orientierung
   4. Übung wechseln
      1. Haben Sie diese Funktion verwendet?
   5. Übung überspringen
      1. Haben Sie diese Funktion verwendet? Wenn ja, welche Gründe gab es für ein Überspringen?
   6. Angabe des Schmerz- und Anstrengungsempfindens nach jedem Durchgang einer Übung? Wren diese hilfreich für Sie?
2. Würden Sie sich weitere Funktionen wünschen? Wenn ja, welche?

Orthese (nur Gruppe AB)

1. Wie empfanden Sie das zusätzliche Tragen der Orthese während des Trainings?
   1. Was war positiv? / Was war negativ?
2. Veränderte das zusätzliche Tragen der Orthese Ihr Gefühl z.B. bezüglich der Kniestabilität, des Sicherheitsempfinden während des Übens, der Belastbarkeit des Kniegelenks oder weiteren Faktoren? Wenn ja, welche?

Akzeptanz der Intervention mit der App / Erfahrung durch die Videos und das Erleben des Trainings

Nützlichkeit einer solchen Intervention

1. Wie beurteilen Sie die gesellschaftliche Relevanz/Notwendigkeit einer solchen App?
2. Glauben Sie, dass Sie ein regelmäßiges Training mit der App in Ihren Lebensalltag in Zukunft integrieren könnten? Warum ja/nein?

Sicherheit

1. Wie sicher sind Sie sich, dass Sie die Übungen mit Hilfe der Instruktionsvideos sowie der Kontrolle durch die Sensoren korrekt durchführen konnten (bzgl. der richtigen Bewegungsausführung)?
2. Hatten Sie Sicherheitsbedenken beim Üben mit der App? Wenn ja welcher Art?
3. Was verstärkte dieses Sicherheitsgefühl? / Was schwächte dieses Sicherheitsgefühl ab?
4. In welchen Bereichen hätten Sie sich eine zusätzliche Hilfestellung gewünscht?

Motivation

1. Wie motiviert waren Sie, die Übungen alleine mit der App zu Hause durchzuführen?
2. Hat Ihnen die App geholfen Ihre Trainingsmotivation zu steigern? Wenn ja, inwiefern?

# Programm

# Umfang/Zeit

1. Wie beurteilen Sie den Umfang der Trainingseinheiten?
2. Wie beurteilen Sie den zeitlichen Aufwand, den Sie für die Ausführung des Trainings mit der App benötigt haben inklusive Anlegen und Kalibrieren der Sensoren?

Inhalte

1. Wie beurteilen Sie die Auswahl der Übungen?
   1. Hinsichtlich der Schwierigkeit der Übungen (ggf. Hilfe: zu einfach / genau richtig / zu schwer)?
   2. Gab es Übungen die Sie nicht durchführen konnten? Aus welchem Grund?
   3. Waren alle Übungen mit den Materialien, die Ihnen zu Hause zur Verfügung standen, durchführbar?
2. Denken Sie an die Trainingsvorgaben (Häufigkeit: 3x/Woche, Wiederholungszahl: bei Kräftigungsübungen 2x25 Wiederholungen in den Wochen 1-6 und 3x15 Wiederholungen in den Wochen 7-12, im Verlauf der 12 Wochen intensiver werdende Übungen). Wie beurteilen Sie die Belastungsdosierung?
   1. Wie beurteilen Sie die an Sie gestellten Trainingsanforderungen in Bezug zu Ihrem Leistungsniveau (ggf. über- unterfordert, genau richtig)?
3. Haben Sie auch die Anweisung für das Training mit dem anderen Bein befolgt und die Übungen dort ebenfalls durchgeführt?

**Umsetzung und Machbarkeit**

1. Welche Erwartungen hatten Sie an die Teilnahme im Vorfeld Studie?
   1. Welche Erwartungen konnten nach den 12 Wochen Training erreicht werden?
   2. Welche Erwartungen wurden nicht erfüllt?

# Vorkommnisse während der Studienzeit

Adverse Events (unerwünschtes Ereignis)

1. Können Sie sich an eine Situation während des Trainings erinnern, in der Sie sich gesundheitlich nicht wohl gefühlt haben (ggf. Hilfe Beschwerden/Schmerzen sich verstärkt haben)?
   1. Wann und in welcher Situation (z.B. in Zusammenhang mit einer bestimmten Übung) trat das Ereignis auf?
2. Können Sie sich an eine Situation während des Trainings erinnern, in der Sie sich gesundheitlich besonders wohl gefühlt haben (ggf. Hilfe Beschwerden/Schmerzen die sich verbessert haben)?
   1. Wann und in welcher Situation (z.B. in Zusammenhang mit einer bestimmten Übung) trat das Ereignis auf?

# Fazit und Praktikabilität für spätere Umsetzung

1. Würden Sie die App in Zukunft gerne nutzen?
2. Was wären Sie bereit für ein solches 12-wöchiges Trainingsprogramm inklusive Sensortechnik zu zahlen?
3. Würden Sie die App weiterempfehlen?
4. Wenn Sie etwas an der App verbessern könnten, damit Sie sie weiterhin nutzen, was wäre das?
5. Gibt es noch etwas, das Sie hinzufügen möchten?

Dank und Verabschiedung

Vielen Dank für Ihre Zeit und Ihre Teilnahme am Interview! Damit ist Ihre Teilnahme an der Studie jetzt beendet. Wir werden Sie über die Studienergebnisse informieren, sobald diese vorliegen. Wir hoffen das Training hat Ihnen Spaß gemacht und die gewünschten körperlichen Erfolge gezeigt und wünschen Ihnen für die Zukunft alles Gute!
