# Supplementary material for: Patients’ experiences and usability of a self-directed m-health exercise intervention for knee osteoarthritis: a qualitative study
Source: BMJ Open. 2025 Jun 16;15(6):e100608. doi: 10.1136/bmjopen-2025-100608 (PMC12314823; doi:10.1136/bmjopen-2025-100608)
Supplement: online supplemental file 2 [file bmjopen-15-6-s002.docx]

| **Appendix 2: Themes, sub-themes, and exemplary quotes.** | |
| --- | --- |
| **(Sub)theme** | **Exemplary quote** |
| **Theme 1: Facilitators for (correct) digital exercise participation** | |
| App features for guidance, support and monitoring of correct exercise execution | AT58: “[…] I thought that was good. The videos were very clear, even from different perspectives, from the side, from the front and so on, you could actually grasp the position, how to stand, how to hold your arms and so on, very well. Additionally, the text description was certainly helpful, as it simply explained a few more details of what to look out for.”  AT38: “That's [the movement bar to display range of motion] good, because of course you have good control over whether you're doing it correctly. You are happy when you hear that clicking sound. And vice versa, you also notice when it is missing. You know, okay, what I'm doing right now isn't perfect and then you can actually make a bit of an effort to do it better and more correctly next time.”  AT51: “[…] the app also corrected you, so that if you didn't do it correctly, you were told to ‘please stretch’ [laughs], so from that point of view, you were corrected. […] I like that feedback, […], you don’t want to be criticized, but it shows you that you are not doing it in the correct way, […]. I thought that was good. |
| Flexibility | AT51: “[…] the advantage is that you don't have to leave the house, you don't have to get changed [your clothes], so I've always dressed sporty of course [laughs], shorts. […], you can do it from home. […]. And you can do it at any time of day.”  AT66: “You could use the app all day, you could get out again, you could get back in again and you simply weren't as time-bound as when you say, okay, I have a fixed appointment at a fixed location.” |
| App features to increase motivation and regularity of the exercise program | AT01: “In doubt, you become more motivated, I would say. And the fact that you also feel a bit monitored by the app certainly increases your level of ambition compared to doing it for yourself or turning pages or something like that. That's how I felt. A certain ambition comes with it.”  AT10: “And it was also fun doing it. I say yes, of course you don't necessarily feel like doing it every day or every second day [laughs], I would rather sit down or do something else. […]. The app reminds you, so you really do have the urge, the compulsion to say ’okay, you have to do sport today or at least a little bit’.”  AT63: “I find it much easier to do this regular training simply because of the support from the app or the knowledge that the app is there and it records it [the training], than it would be without the app.”  AT10: “No, I mean in principle I had a very secure feeling. In principle, the avatar made me feel very confident. You can actually see quite well whether you are doing the exercises correctly.” |
| Positive experiences with the exercise program | AT27: “Yes, overall the second half of the training phase was very positive. When I was doing new exercises, I realized that they really weren't that difficult anymore and once I knew the exercises, I noticed how much better they worked and when the update fixed the calibration problem, it was really fun.”  AT56: “You don't need weights to exercise in order to make progress. I would never have thought that, if it is only to hold the exercise accordingly, i.e. some exercises require you to hold the position for three seconds, three, two, one, you would not believe how long three seconds are. Yes, it was really good.”  AT04: “I mean, I thought it was good that you had two exercises that were relatively easy most of the time and two exercises that were a bit difficult [in every exercise session]. That’s how I felt, and then one exercise was for stretching or relaxation. […] that’s very good, that’s fine, you can do it, you can manage it.”  AT58: “The amount. Yes, the individual sessions, as I said, that's good, it can be integrated, it's achievable, even if you are not really motivated, you know it will be over in half an hour [laughs] and the 12 weeks, yes, that's a reasonable length. I don't think I have ever done any exercises so consistently for 12 weeks.” |
| **Theme 2: Barriers for (correct) digital exercise participation** | |
| No on-site instructions by a health professional | AT27: “Obviously, a physiotherapist can see even more. The sensors have controlled my leg and when I do the exercises with a physiotherapist, they can of course also see the rest of my body and whether something is going wrong in other parts of the musculoskeletal system, e.g. that you assume a bad posture elsewhere in order to do the knee exercise. Of course, the app can't see that, it can only see the movement of the knee.”  AT07: “So that I couldn’t show someone on site how I do it [the exercises], and then they say to me, ‘you overlooked the fact that you should have done this or that’. Then it might have been easier for me to accept that the app was right.” |
| Technical issues | AT56: “Sometimes it was annoying because the nice female voice always pointed out ‘bend your knee more’ or when you had the exercise band in a hip extension movement, in which direction should I […] I have to say it differently the advice came ‘stretch more, a bigger lunge’, sometimes more [range of motion] was not possible at all. I couldn’t get rid of the error.”  AT58: “I have to say that the correction factor was very difficult to implement for certain exercises. These include exercises with the band, for example, where you move your leg sideways, outwards, abduction I think it’s called. […] I just couldn’t complete the exercise, because something was always wrong, even though I couldn’t go any further [regarding the range of motion]. […] So, it was always ‘keep going outwards’, but it just didn’t go any further and at some point, you give up. I think I stopped the exercise once or twice.  AT27: “Yes, I skipped certain exercises as I was always asked to recalibrate because the device didn't recognize what my leg was doing and the avatar looked really dangerous and my virtual knee bent sideways. Then there were no useful hints and then I stopped some exercises, in total I stopped four exercises or something like that. Not the whole workout, but certain exercises.” |
| Difficulties with the exercise program | AT63: “Well, I mean, of course there are certain exercises that you like to do and other exercises you tend to say no to. So, for me, for example, all those squat exercises that involved doing knee flexion were always rather disliked because they simply caused pain in my knee.”  AT38: ”I actually only had, I think, one flexion exercise where I actually thought ’I don't like this right now or my knee doesn't like this right now‘. Usually, that are always exercises that require a lot of flexion and load on the affected leg. A lot of standing on the affected leg, that is actually something where I just have some problems.”  AT58: “That was this downward stair exercise and a weight-bearing exercise on the damaged knee where you had to shift to the front. It was always difficult when a load was combined with a downward movement.”  AT56: “That was the exercise on the wall. That was the pushing up and down, exactly. Because it was very demanding for my knees.”  AT07: “[…], what was very difficult for me personally, at that time I had relatively severe pain in my knees, was the fact that exercises on the floor were prescribed. So, for me, these exercises meant that I first had to get down on the floor and then come up again afterwards. […] that was uncomfortable for me.” |
| Associated problems with app features | AT38: “So then I just, I just kept trying until something happened and then I don't think the exercise was done exactly the way it was supposed to be. It was a bit bizarre, because then it was obvious that I wasn't doing it the way I was supposed to, but then I kind of wanted to, I wanted to make it count.”  AT56: “Good, good. As I said, it was annoying when the error occurred several times. Sometimes I would have preferred to switch off the voice or whatever, but apart from that it was easy to understand. The error was there, you try to fix it and I got to deal with it quite quick, if that is what happened.”  AT53: “Yes, I did it, but sloppily. Because I just didn't have had the motivation, as I said, the sensor on the other leg would be very useful. You do it faster there, you don't hold the time as long, because you have to count it down yourself, and it's just, yes, you just don't do it. So, I don't.” |
| **Theme 3: Ease of use** | |
| Usability, feasibility and comprehensibility | AT27: “Putting on these sensors, starting the app and things like that, you get into a routine rather fast, so I actually found it to be very smooth and good.”  AT58: “ […] that was easy to understand, most of it was easy to understand and to implement.”  AT56: “In general, the performance when using the software, for me it was easy to understand, I got to grips with it quite fast and that counts for me when I get to grips with an IT device or software rather fast.”  AT38: “Of course you have to find your way around it, you have to see what the individual menu items offer and where to go. But it's so good and self-explanatory that I think you can work with it without any major problems.”  AT10: “It is quick and easy. It is very quick, very easy. So, I found that it was no problem at all for me. You put on the straps, put on the sensors, open the app and then get started [laughs].” |
| Handling and screen size | AT10: “Sometimes it [smartphone] was just too small for me. But of course, as soon as you have either a computer in front of you, a monitor or a tablet, then of course it is better.”  AT57: “Well, of course, the handling of the iPad was sometimes difficult, because when you're lying on the floor, where do I put it, where do I hang it up? [laughs] Well, it was good at the table, I could, my wife gave me a reading stand, a book stand, it was quite good, you could do it there, but if you're lying on the floor, it's difficult of course.” |
| **Theme 4: Outcomes** | |
| Positive Outcomes | AT51: “[…] the muscles around the knee were definitely strengthened, which I probably wouldn’t have done in that time without this app.”  AT27: “[...] in my case it relieved the symptoms, and increased flexibility [...]”  AT53: “But I think I can walk down the stairs very quickly with both legs, I only walked down the stairs with one foot step-by-step, the other foot following, that's how I walked down the stairs. Now I can walk down the stairs with both legs at a brisk pace, which is a huge success.”  AT53: “The pain has also improved a lot, a lot, it is not gone. But, I didn’t expect that either.”  AT56: “There has been a significant increase in strength of my thigh muscles, because now I can also feel it on the bike. It is at least one gear easier.”  AT27: “That was actually what I expected - better flexibility, better stability and being able to do more things without pain - and they [the expectations] were fulfilled. For example, I rode my bike up to the health center for the final consultation, which is something I couldn't have imagined in October or even in January that it would be possible, in terms of the strain on my knee, and it was easy to do. As an example of how much this has made my knee usable again.”  AT38: “I have the feeling that it's up to me what I do with it. It puts me in a position where I can just keep at it [doing exercises].” |
| Not met outcome expectations | AT10: “I don't have the feeling that I really have more strength in my legs because of it, so I haven't noticed any difference.”  AT58: “As I said, the expectation that it will really improve to such an extent that surgery is no longer an option is not given, so that I will probably expect one in the next six or nine months. I didn't really have had any other expectations.”  AT07: “No. For the simple reason that at some point I stopped enjoying it. So, I didn't have had any medical, I didn't have had the feeling that I was somehow [pause] building up I don't know what skills, I didn't have had any experience of success, that it had improved or that I was better at it or whatever.” |
