# Supplementary material for: Patients’ experiences and usability of a self-directed m-health exercise intervention for knee osteoarthritis: a qualitative study
Source: BMJ Open. 2025 Jun 16;15(6):e100608. doi: 10.1136/bmjopen-2025-100608 (PMC12314823; doi:10.1136/bmjopen-2025-100608)
Supplement: online supplemental file 3 [file bmjopen-15-6-s003.pdf]

# Appendix III: Study protocol

Universitätsklinikum Tübingen

**Prüfplan zur Studie mit dem Titel:**

Randomisiert-kontrollierte Studie im Wartelisten-Kontroll-Design zur Überprüfung einer 12-wöchigen app- und orthesengestützten Trainingsintervention bei Patienten mit mittel- bis schwergradiger Gonarthrose

| | |
|---|---|
| Datum des Prüfplans: | 01.07.2020, Ergänzungen vom 27.08.2020 unter Berücksichtigung der Rückmeldung der Ethikkommission vom 17.08.2020 |
| Institution: | Universitätsklinikum Tübingen (UKT)<br>Medizinische Klinik, Abteilung Sportmedizin<br>Hoppe-Seyler-Str. 6<br>72076 Tübingen |

Anlagen:

1 Probandeninformation

2 Einverständniserklärung

3 CE-Kennzeichnung GENUDYN®OA smart (Firma Sporlastic) und re.flex-Technologie (Kineto Tech Rehab SRL)

# Inhaltsverzeichnis

1 Einleitung ... 3
2 Ziele der Studie ... 5
3 Material und Methode ... 5
3.1 Studiendesign ... 5
3.2 Studienpopulation ... 5
3.3 Fallzahl ... 7
3.4 Probandenrekrutierung ... 7
3.5 Abbruchkriterien ... 8
3.6 Studienablauf ... 8
3.7 Randomisation ... 11
3.8 Trainingsintervention ... 11
3.9 Zielmessgrößen ... 14
3.9.1 Patientencharakteristika ... 18
3.9.2 Patientenberichtete Endpunkte ... 18
3.9.3 Kraftdiagnostik ... 19
3.9.4 Gleichgewichtstest ... 19
3.9.5 Patientensicherheit ... 20
3.10 Datenanalyse ... 21
4 Zeitplanung ... 22
5 Datenschutz ... 23
5.1 Probandenkennung und Datenbank ... 23
5.2 Wahrung der ärztlichen Schweigepflicht ... 23
6 Versicherungsschutz ... 23
7 Vor- und Nachteile für Probanden / Risiko ... 23
8 Interessenskonflikt ... 24
Literaturverzeichnis ... 25
Anlagen ... 27

# 1 Einleitung

In Deutschland sowie weltweit gilt die Arthrose als die am häufigsten vorkommende degenerative Gelenkerkrankung. Dabei kommt es zu einer zunehmenden Zerstörung des Gelenkknorpels bis hin zur Freilegung der Knochenoberfläche und auch die gelenkumgebenden Strukturen sind von dem Krankheitsbild betroffen. Die Studie GEDA 2014/2015-EHIS des Robert Koch Instituts zeigt eine 12-Monats-Prävalenz von 17,9 % der Erwachsenen ab 18 Jahren in Deutschland, die vom Vorliegen einer Arthrose betroffen sind. Mehr als die Hälfte der Betroffenen leiden dabei an einer Gonarthrose, also einem Befall des Kniegelenks. Neben einem steigenden Lebensalter zählen auch das weibliche Geschlecht, Über- und Fehlbelastung der Gelenke (z.B. Achsfehlstellungen), Verletzungen und Unfälle oder Übergewicht zu den möglichen Risikofaktoren bzw. Mitursachen für Arthrose [1]. Mit zunehmendem Schweregrad der Gonarthrose werden häufiger Schmerzen, Einschränkungen der körperlichen Funktionsfähigkeit sowie eine verminderte gesundheitsbezogene Lebensqualität seitens der Patienten berichtet. Als konservative Therapieform wird von den nationalen und internationalen Leitlinien die Sport- und Bewegungstherapie empfohlen [2]. Da es sich um eine degenerative Erkrankung handelt, ist das primäre Ziel die Beschwerdesymptomatik zu lindern, die Funktionsfähigkeit zu verbessern sowie das Selbstmanagement und eine Wissensvermittlung zu fördern. Mechanisch gesehen, soll dabei die Stabilisierung der Umgebung des Gelenks verbessert sowie eine unphysiologische Gelenkbelastung reduziert werden [3]. Bei entsprechend trainingswirksamer Belastungsdosierung zeigten verschiedene Trainingsformen zur Muskelkräftigung sowie Verbesserung der Gleichgewichtsfähigkeit und Beweglichkeit zumindest kurz- und mittelfristig einen positiven Behandlungseffekt [4,5]. Es scheint aber weiter Bedarf an der Entwicklung eines Programms zu geben, das zur langfristigen Erhaltung eines regelmäßig durchgeführten Übungsprogramms motiviert [5].

Neben land-basierten Therapieangeboten in Gruppen oder physiotherapeutischer Behandlung, spielt für die langfristige Aufrechterhaltung der Sport- und Bewegungstherapie auch das heimbasierte, eigenständig durch den Patienten durchführbare Training eine wichtige Rolle [3]. Dabei können in Zukunft vor allem auch digitale Applikationen (Apps) eine Möglichkeit bieten, die Patienten in der korrekten und sicheren Übungsausführung zu unterstützen. Zudem soll die App zur Anwendung einer kontrollierten Belastung sowie zum Umgang mit möglicherweise auftretenden Schmerzen anleiten. Die Vorteile der digitalen Bereitstellung eines Therapieprogramms liegen dabei primär in der räumlich und zeitlich unabhängigen Durchführbarkeit und großen Reichweite eins möglichen Nutzerkreises [6,7].

Auch orthopädische Hilfsmittel, beispielsweise in Form von Knie- und Unterschenkelorthesen oder Einlagen, werden in den Leitlinien zur Gonarthrosetherapie bereits diskutiert [2]. Diese

versuchen bei unikompartimentellem Befall, den entsprechenden Bereich zu entlasten und eine Verlagerung der Belastung auf nicht oder bedeutend weniger betroffene Gelenkkompartimente zu erzeugen sowie das Gelenk zu stabilisieren. Dies spielt insbesondere bei Achsfehlstellungen eine Rolle. Bisher wurden überwiegend Untersuchungen bei medialer Gonarthrose, die meist mit einer varischen Beinachse einhergeht, durchgeführt [8,9]. Zum Teil liegen dabei bereits positive Wirksamkeitsnachweise bezüglich einer Schmerzreduktion und Funktionsverbesserung sowie einer Verbesserung der Gelenkmechanik vor [9]. In weiterführenden Studien müsste untersucht werden, ob ein komplementärer Einsatz einer Orthese zur Sport- und Bewegungstherapie möglicherweise sogar einen zusätzlichen positiven Wirkungseffekt erzielen und die körperliche Aktivität dadurch weiter gesteigert werden kann.

Am 19. Dezember 2019 wurde mit dem Inkrafttreten des Digitale-Versorgungs-Gesetzes (DVG) die „App auf Rezept“ in die Gesundheitsversorgung eingeführt. Die gesetzliche Krankenversicherung (GKV) umfasst ca. 73 Millionen Versicherte, die damit einen Anspruch auf digitale Gesundheitsanwendungen (DiGA) haben. Eine DiGA wird zur Unterstützung der „Erkennung, Überwachung, Behandlung oder Linderung von Krankheiten“ oder der „Erkennung, Behandlung, Linderung oder Kompensierung von Verletzungen oder Behinderungen“ eingesetzt [10]. Sie werden durch die Krankenkassen erstattet. Eine DiGA wird in der Regel von Ärzten verordnet, kann aber bei entsprechend vorliegender Indikation auch ohne ärztliche Verordnung erhalten werden. Die DiGA muss dafür ein Prüfverfahren des Bundesinstituts für Arzneimittel und Medizinprodukte (BfArM) bestehen und in einem neu geschaffenen DiGA-Verzeichnis gelistet werden. Teil der Prüfung ist hierbei ein durch den Hersteller zu erbringender Nachweis eines positiven Versorgungseffektes, der mit der DiGA einhergeht. Unter einem positiven Versorgungseffekt wird dabei ein medizinischer Nutzen oder eine patientenrelevante Struktur- und Verfahrensverbesserung in der Versorgung verstanden. Es muss mindestens ein positiver Effekt aus mindestens einem der genannten Bereiche bestehen. Ein medizinischer Nutzen liegt vor, wenn eine Verbesserung des Gesundheitszustands, eine Verkürzung der Krankheitsdauer, eine Verlängerung des Überlebens oder eine Verbesserung der Lebensqualität erzielt werden kann. Die patientenrelevanten Struktur- und Verfahrensverbesserungen beziehen sich insbesondere auf die folgenden Bereiche: Koordination der Behandlungsabläufe, Ausrichtung der Behandlung an Leitlinien und anerkannten Standards, Adhärenz, Erleichterung des Zugangs zur Versorgung, Patientensicherheit, Gesundheitskompetenz, Patientensouveränität, Bewältigung krankheitsbedingter Schwierigkeiten im Alltag und Reduzierung der therapiebedingten Aufwände und Belastungen der Patienten und ihrer Angehörigen. Zu den Anforderungen des Nachweises der positiven Versorgungseffekte gehören die Ergebnisse

einer quantitativen Vergleichsstudie, z.B. einer randomisierten kontrollierten Studie (RCT), die eine Überlegenheit der DiGA-Anwendung gegenüber einer Nichtanwendung zeigt [10].

# 2 Ziele der Studie

Primäres Ziel der geplanten Studie ist die Untersuchung der Wirksamkeit und Unbedenklichkeit einer 12-wöchigen app-gestützten Trainingsintervention (Gruppe Non-Waitlist-Control, NWLC) im Vergleich zu einer Kontrollgruppe (Waitlist-Control, WLC) ohne Intervention. Die Wirksamkeit soll sowohl für die alleinige Nutzung einer App (Gruppe A, app-gestütztes Training), als auch in Kombination mit der Nutzung einer Knieorthese (Gruppe OA, orthesen- und app-gestütztes Training) überprüft werden.

Dabei werden die Erbringung eines positiven Versorgungsnachweises und die damit verbundene Eintragung in das DiGA-Verzeichnis angestrebt.

# 3 Material und Methode

## 3.1 Studiendesign

Die Studie soll im randomisierten Wartelisten-Kontroll-Design mit zwei Interventionsgruppen (A und OA) durchgeführt werden. Die Studie wird von der Abteilung Sportmedizin des Universitätsklinikum Tübingen durchgeführt.

## 3.2 Studienpopulation

Die Studienpopulationen setzt sich aus Probanden mit mittel- bis schwergradiger medialer oder lateraler Kniearthrose zusammen. Folgende Ein- und Ausschlusskriterien werden weiter berücksichtigt (siehe Tabelle 1).

**Tabelle 1: Ein- und Ausschlusskriterien zur Studienteilnahme.**

| Einschlusskriterien | Ausschlusskriterien |
|---|---|
| - Alter ab 18 Jahren | - Termin für einen elektiven Ersatz des Kniegelenks bzw. vorhandene Knie-TEP im Signalgelenk[1] |
| - Signalgelenk: betroffenes Kniegelenk | - Signalgelenk Hüfte o. ä.<br>- diffuse Kniebeschwerden, retropatellare Kniearthrose oder beidseitige (medial |

[1] Das Signalgelenk beschreibt das betroffene, symptomatische Gelenk. Werden beispielsweise Schmerzen in Hüfte und Knie oder beiden Knie angegeben, ist das Signalgelenk jenes das stärker betroffen ist/ mehr Schmerzen hervorruft.

| | |
|---|---|
| - bei beidseitiger Symptomatik das Knie mit der stärkeren Symptomatik (Zeitfenster 4 Wochen)<br>- einseitige Kniebeschwerden (medial oder lateral) | und laterale) Beschwerdesymptomatik als Kontraindikation zum Tragen einer korrigierenden Orthese |
| - Diagnose einer mittel- bis schwergradigen medialen oder lateralen Kniearthrose:<br>1. Haben Sie aufgrund von Beschwerden im Kniegelenk schon einmal einen Arzt aufgesucht?<br>2. Wurde bei Ihnen jemals von einem Arzt eine Arthrose oder eine degenerative Gelenkerkrankung an den Knien festgestellt (Arthrose bedeutet „Gelenkverschleiß)?[2]<br>→Frage 2 muss zum Einschluss mit „JA" beantwortet werden<br>- Mündliche Abfrage der Subskala Schmerz des Knee injury and Osteoarthritis Score (KOOS) → KOOS Schmerz Score ≤60 | - Erkrankungen, die die Belastungsfähigkeit im Alltag beeinträchtigen (z.B. schwerwiegende Herz-Kreislauf-Erkrankungen): Abfrage über Erhebungsbogen in Anlehnung des PAR-Q |
| - Ärztliche Untersuchung zur Diagnosesicherung sowie Indikation und Tragemodus der Valgus-/Varus-Orthese | - Erkrankungen der unteren Extremität oder des unteren Rückens, aufgrund derer der Patient derzeit in ärztlicher oder therapeutischer Behandlung ist |
| - Gute Deutschkenntnisse (Verständnis der Probandeninformation, der eingesetzten Fragebögen und der Anleitungen der App) | - vorausgehende Operationen, Verletzungen oder Erkrankungen, die die Kraftmessungen, Messungen des Gleichgewichts und eine Trainingsintervention beeinträchtigen können |
| - Bereitschaft eine Orthese zum Training und ggf. im Alltag zu tragen | - |

[2] Befragung In Anlehnung an den Fragebogen zur Studie „Gesundheit in Deutschland aktuell" GEDA 2014/2015-EHIS des Robert Koch-Instituts [11]

| | |
|---|---|
| - Bereitschaft zum app-gestützten Training (Vorhandensein eins Endgerätes mit dem Betriebssystem iOS) | - Android-Betriebssystem |
| - Sporttauglichkeit (siehe Erhebungsbogen in Anlehnung an PAR-Q[3]) | |
| - Einwilligung zur Studienteilnahme | |

## 3.3 Fallzahl

Die Fallzahlschätzung der vorliegenden Studie wurde auf Grundlage einer a priori Poweranalyse durchgeführt (G*Power 3.1.9.6). Hierzu wird der Innersubjektfaktor*Zwischensubjektfaktor Interaktionseffekt einer 2 x 2 ANOVA mit Messwertwiederholung zugrunde gelegt. Die Stufen des Zwischensubjektfaktors (Gruppe) sind hierbei 1.) die „Waitlist-Control“ (WLC), 2.) die zusammengefassten Interventionsgruppen der „Non-Waitlist-Control“ (NWLC). Die der Fallzahlschätzung zugrundeliegende Fragestellung bezieht sich also auf App versus Kontrolle über die Zeit – unabhängig von der Art der Intervention (nur App (A) oder in Kombination mit der Orthese (OA)). Die Level des Innersubjektfaktors sind die Erhebungszeitpunkte t0 (prä) und t1 (post). Der Schätzung liegen zudem vorliegende Annahmen zugrunde: Alpha-Niveau $\alpha$=0.05, Teststärke (Power) $\beta$=0.8 sowie ein Zusammenhang zwischen Prä- und Postmessungen von r=0.5. Aufgrund fehlender Daten aus Studien mit vergleichbaren Interventionen wurde auf standardisierte Effektstärken zurückgegriffen und der Bereich von f=0.20 (entspricht einem Cohen’s d = 0.4) bis f=0.4 (entspricht einem Cohen’s d = 0.8) berechnet. Unter Berücksichtigung der oben genannten Annahmen ist eine Gesamtfallzahl von 52 (f=0.20) bis 20 (f=0.4) erforderlich (n=17/Gruppe). Unter Annahme einer mittleren Effektstärke und einer Drop-out-Rate von ca. 15% sollen n=30 Personen je Gruppe (WLC, NWLC) rekrutiert werden, um einen Effekt von f=0.20 nachweisen zu können.

## 3.4 Probandenrekrutierung

Potentielle Probanden werden über Zeitungsannoncen, Flyer und Rundmails (UKT und Universität Tübingen) rekrutiert.

[3] Physical Activity Readiness Questionnaire. Der PAR-Q-Test ist ein Fragenkatalog, mit dem sich auf einfache Weise feststellen lässt, ob aus medizinischer Sichtweise grundsätzlich Bedenken bei der Ausübung von körperlichen Aktivitäten bestehen. Er wurde von der Canadian Society for Exercise Physiology entwickelt. Sein Einsatz wird im Vorfeld der Aufnahme einer körperlichen Aktivität auch von der Deutschen Gesellschaft für Sportmedizin und Prävention e. V. (DGSP) empfohlen [12,13].

Die Probandenrekrutierung erfolgt in einem zweistufigen Verfahren. Die erste Kontaktaufnahme erfolgt per Telefon oder Email und dient der Information der Probanden über die Studieninhalte und –ziele sowie den zeitlichen Studienablauf. Zudem erfolgt dabei das erste Screening zur Prüfung der Ein- und Ausschlusskriterien. Zur Abklärung der Sporttauglichkeit wird zusätzlich ein Erhebungsbogen (in Anlehnung an den PAR-Q mit ergänzenden orthopädischen Fragen) durchgeführt. Können nicht alle Fragen mit einem „ja" beantwortet werden, so kann eine Teilnahme an der Studie nur gewährt werden, wenn die Sporttauglichkeit für die Übungstherapie durch einen Arzt erteilt wurde. Die ärztliche Untersuchung zur Sporttauglichkeit ist nicht Teil der Studie und wird bei Interesse eines Patienten durch diesen eigenständig veranlasst und wahrgenommen.

Bei Studieneignung und Interesse seitens der Probanden wird in einem zweiten Schritt ein Termin für den ersten persönlichen Kontakt vereinbart. Dieser Termin beinhaltet eine mündliche und schriftliche Aufklärung und die Beantwortung offener Fragen. Nach schriftlicher Einwilligung des Probanden (siehe Probandeninformation und Einwilligungserklärung) gilt dieser als vorläufig eingeschlossen. Im Anschluss erfolgt eine ärztliche Eingangsuntersuchung (Anamnese und körperliche Untersuchung). Ergeben sich aus Sicht des Arztes keine weiteren Einschränkungen, so gilt der Proband als endgültig eingeschlossen.

## 3.5 Abbruchkriterien

Im Rahmen der Einverständniserklärung wird der Proband explizit darauf hingewiesen, dass die Teilnahme an der Studie freiwillig ist und jederzeit und ohne Angabe von Gründen beendet werden kann. Es wird ferner darauf hingewiesen, dass die Studienleitung bei vorliegendem triftigem Grund (z.B. gesundheitliche Gefährdung des Patienten bei Fortsetzung der Studie) den Proband von der weiteren Teilnahme an der Studie ausschließen kann.

## 3.6 Studienablauf

Die Studiendauer für den einzelnen Probanden beträgt 27-28 Wochen. Zu Beginn wird eine orthopädische Eingangsuntersuchung zur Diagnosesicherung einer medialen oder lateralen Gonarthrose, zur Abklärung möglicher Kontraindikationen bezüglich des Tragens einer Orthese bzw. der Durchführung des Trainingsprogramms sowie der Orthesenauswahl und des Tragemodus (Valgus-/Varus-Orthese) durchgeführt. Es folgen die erste Datenerhebung im Zeitraum von zwei Woche (Testbatterie t0), die 12-wöchige Interventions- (NWLC) bzw. Wartephase (WLC) und eine erneute Datenerhebung t1 im Zeitraum einer Woche, die 12-wöchige Interventions- (WLC) bzw. Follow-up-Phase (NWLC) und einer abschließenden Messung in der 28. Studienwoche (t2). Die Testbatterie beinhaltet jeweils isometrische Maximalkraftmessungen der unteren Extremität, die Messung der posturalen Stabilität sowie die Erfassung der studienrelevanten Fragebögen (Selbstauskunft). Details zu den

Messverfahren und Messgrößen sind dem Kapitel 3.9 zu entnehmen. Bei der Intervention handelt es sich um ein app-gestütztes und inertialsensor-kontrolliertes 12-wöchiges gonarthrosespezifisches Trainingsprogramm. Eine Gruppe führt das Training ohne weitere Ergänzungen durch (A), eine weitere Gruppe erhält für Training und Alltag zusätzlich eine Knieorthese (OA). Details zur Intervention sind dem Kapitel 3.8 zu entnehmen. Das Randomisationsverfahren zur Zuteilung der Studienprobanden in WLC und NWLC sowie A und OA wird im folgenden Kapitel 3.7 erläutert. Nach Zuordnung der Interventionsgruppen folgt für die Gruppe OA eine Anpassung der Knieorthese (GENUDYN®OA smart, Sporlastic) durch einen ausgebildeten Orthopädietechniker der Kooperationsfirma Sporlastic GmbH.

Der Studienablauf ist übersichtlich in Abbildung 1 zusammengefasst.

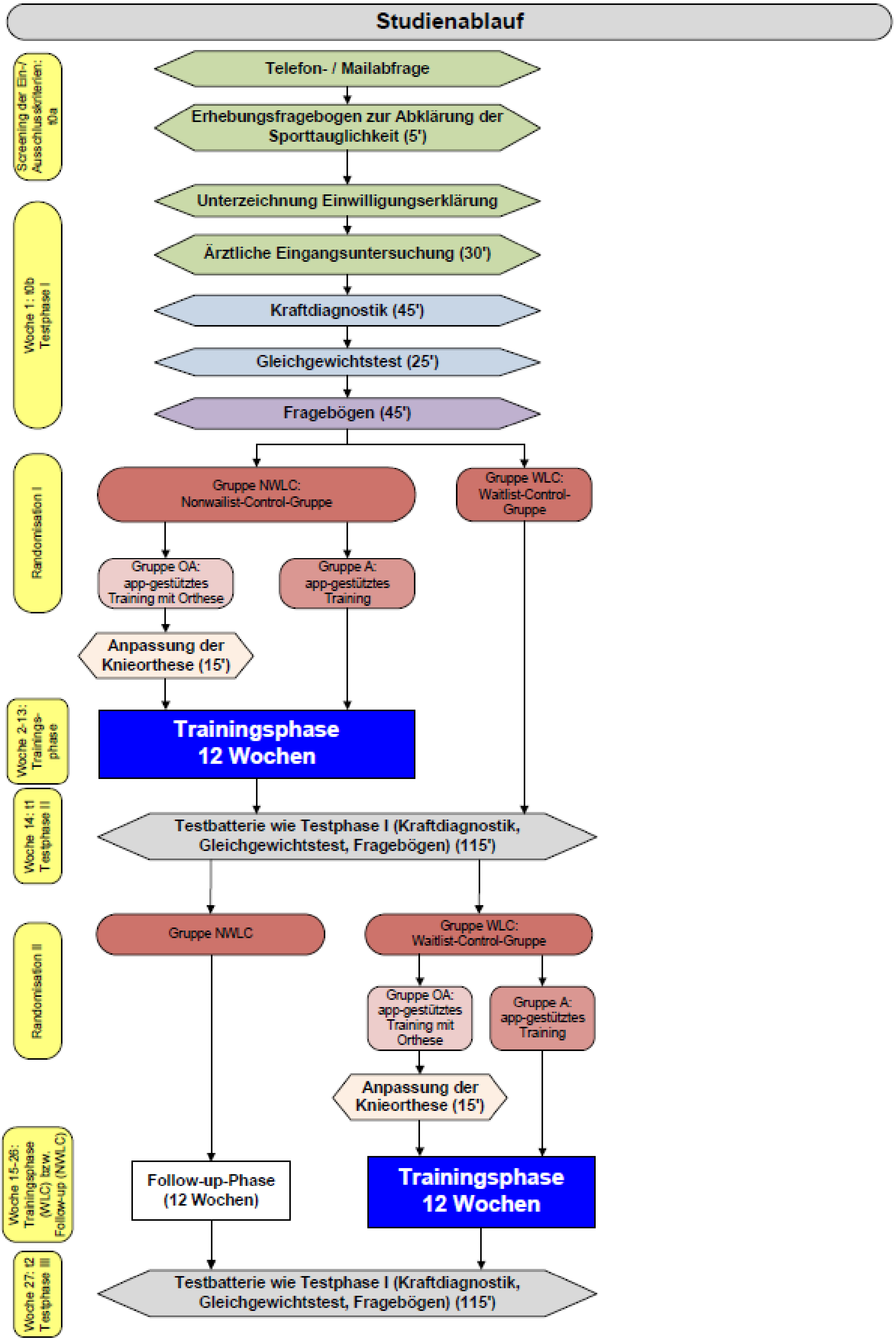


**Abbildung 1: Studienablauf.**

## 3.7 Randomisation

Die Randomisationsliste wird vor Beginn der Studie in Excel (Funktion Zufallsbereich (0;1) in 7 Blöcken mit je 10 Slots) erstellt. Im Anschluss werden versiegelte Couverts mit einer fortlaufenden Nummer versehen, die entsprechend der Liste die Gruppenzuteilung für die fortlaufenden Nummern beinhaltet. Die Erstellung der Randomisationsliste und die Versieglung erfolgt durch einen Mitarbeiter, der ansonsten nicht in die Studiendurchführung und Studienauswertung involviert ist. Die Zuteilung der Probanden in die Gruppe WLC bzw. NWLC erfolgt in der Reihenfolge ihrer Einbestellung nach erfolgreicher Testung zu t0 durch Öffnung des mit der kleinsten fortlaufenden Nummer verbleibenden verschlossenen Couverts. Sollten Probanden vor Abschluss der Eingangstests ausfallen, so entfällt auch die Randomisation. Der für die Tests zuständige Mitarbeiter ist auch für den Prozess der Randomisation zuständig. Probanden der WLC werden zunächst ohne weitere Randomisierung weitergeführt. Patienten der NWLC werden in Analogie des zuvor genannten Verfahrens in einer zweiten Stufe randomisiert einer der beiden Interventionsformen im Verhältnis 1:1 zugeteilt (Gruppe OA: app-gestütztes Training mit Orthese und Gruppe A: app-gestütztes Training). Auch hier erfolgt die Erstellung und Versiegelung der Randomisationslose vor Studienbeginn. Eine erneute Testung t1 erfolgt nach der 12-wöchigen Interventionsphase der NWLC (Post) bzw. der 12-wöchigen Wartephase der WLC (Pre 2). Im Anschluss an diese Tests werden Probanden der WLC einer der beiden Interventionsgruppen OA bzw. A zugelost. Hierbei werden die verbleibenden Couverts aus der Randomisation zu t0 in konsekutiver Reihenfolge der Testtermine vergeben. Auch hier erfolgt die Zuteilung durch den Mitarbeiter, der für die Tests verantwortlich ist. In den folgenden 12 Wochen erfolgt die Intervention für Probanden der WLC und keine weitere Studienintervention für die NWLC.

## 3.8 Trainingsintervention

Die 12-wöchige Trainingsintervention (siehe Abbildung 2) wird in zwei Phasen mit jeweils 6 Wochen durchgeführt. Im Vordergrund steht die Kräftigung der Muskelgruppen Kniestrecker, Kniebeuger und Hüftabduktoren, aber auch Mobilisation, Dehnung und Gleichgewichtstraining sind Trainingsinhalte. Phase I ist gekennzeichnet von einem Kraftausdauertraining, wobei der Schwerpunkt zunächst bei der Gewöhnung an Bewegung und Belastung und dem Kennenlernen und Wahrnehmen der Übungen sowie der korrekten Übungsdurchführung und einer optimalen Belastungsdosierung liegt. Ebenfalls sind die Bewegungserweiterung und Schmerzreduktion wesentliche Trainingsziele. In Phase II wird das Muskelaufbautraining durchgeführt. Hier liegt der Trainingsschwerpunkt im Bereich der Kräftigung. Die Teilnehmer führen die Trainingsintervention je nach Gruppenzuteilung als app-gestütztes Training (re.flex, © 2019, KINETO TECH REHAB SRL) in Kombination mit einer Orthese (GENUDYN®OA smart, Sporlastic) und darin integrierten Beschleunigungssensoren (Gruppe OA) oder als app-

gestütztes Training (re.flex, © 2019, KINETO TECH REHAB SRL) in Kombination mit Beschleunigungssensoren (Gruppe A) durch. Die Orthese bzw. die Sensoren werden jeweils am mit Gonarthrose betroffenen Bein befestigt. Die Orthese selbst sowie die re.flex Technologie inklusive der Beschleunigungssensoren verfügen über eine jeweils eigentständige CE-Kennzeichnung. Die App fungiert dabei als Trainingspartner, der die Übungen inklusive Übungsbeschreibungen und –videos, Wiederholungszahlen und Anzahl an Durchgängen vorgibt. Zudem werden über die Beschleunigungsensoren die durch einen in der App integrierten Avatar vorgegebenen Gelenkwinkel und damit der Bewegungsumfang sowie die Bewegungsgeschwindigkeit der Übungen kontrolliert. So wird eine korrekte Ausführung der Übungen unterstüzt. Das Tracken der Übungen ermöglicht weiter die Aufzeichnung korrekt durchgeführter Wiederholungen und Trainingstage. Anfangs geht es zunächst darum, die Übungen kennenzulernen und korrekt auszuführen sowie den Umgang mit einer optimalen Belastungsdosierung zu erlernen. Im Verlauf sollen die Probanden dazu befähigt werden, eigenständige Anpassungen der Übungsschwierigkeit je nach Anstrengungs- und Schmerzempfinden vorzunehmen. D.h. in Phase I können die Probanden ab Woche 3 immer auch zwischen einer leichteren (Übungsvariante blau) und schwereren (Übungsvariante rot) Übung auswählen. Dabei müssen sie den Grund der Änderung über ein Schmerz-/Anstrengungsprotokoll dokumentieren. Nach und nach kann in den geraden Wochen der Phase II (Woche 8, 10 und 12) zudem beispielsweise auch zwischen Ausgangsstellungen und geschlossener/offener Kette einer Zielmuskelgruppe ausgewählt werden. D.h. die Trainingspläne und Übungen sind nicht mehr im Detail vorgegeben, sondern können beispielsweise über die Ansage: „Wählen Sie aus den Übungskategorien Kniestrecker und Kniebeuger jeweils 1 Übung aus!“ sowie der Wahlmöglichkeit des Schwierigkeitsniveaus (blaut/rot) in Abhängigkeit des Schmerz- und Anstrengungsempfinden selbst mitbestimmt werden.

Insgesamt sind 3-4 Trainingseinheiten mit einer Dauer von jeweils 20-25 min pro Übungswoche geplant. Um eine progressive Steigerung des Therapieprogramms zu erreichen, werden neben verschiedenen Übungen und Übungsvarianten (z.B. im kurzen oder langen Hebel) auch Minibänder zur Verstärkung des Widerstandes als Trainingsmittel zum Einsatz kommen.

Damit keine muskuläre Dysbalance enstehen kann, soll der Trainierende die Übungen auch mit dem Bein ohne Orthese/Sensor durchführen.

Die wichtigsten Trainingsparameter sind in Tabelle 2 zusammengefasst.

**Tabelle 2: Übersicht Trainingsparameter 12-Wochen-Therapieprogramm.**

| | |
|---|---|
| **Gesamtumfang** | 12 Wochen |
| **Anzahl Einheiten pro Woche** | 3-4 |
| **Dauer pro Einheit** | 20-25 |
| **Pausengestaltung** | Training des Bein ohne Orthese bei einbeinigen Übungen<br>45 sec Pause bei beidbeinigen Übungen |
| **Muskulatur** | Kniestrecker, Kniebeuger, Hüftabduktoren |
| **Ausgangsstellungen** | Rückenlage, Sitz, Stand (ggf. mit Abstützhilfe) |
| **Trainingsinhalte** | Kräftigung, Gleichgewicht, Mobilisation, Dehnung |
| **Trainingsmittel** | Stuhl, Miniband |

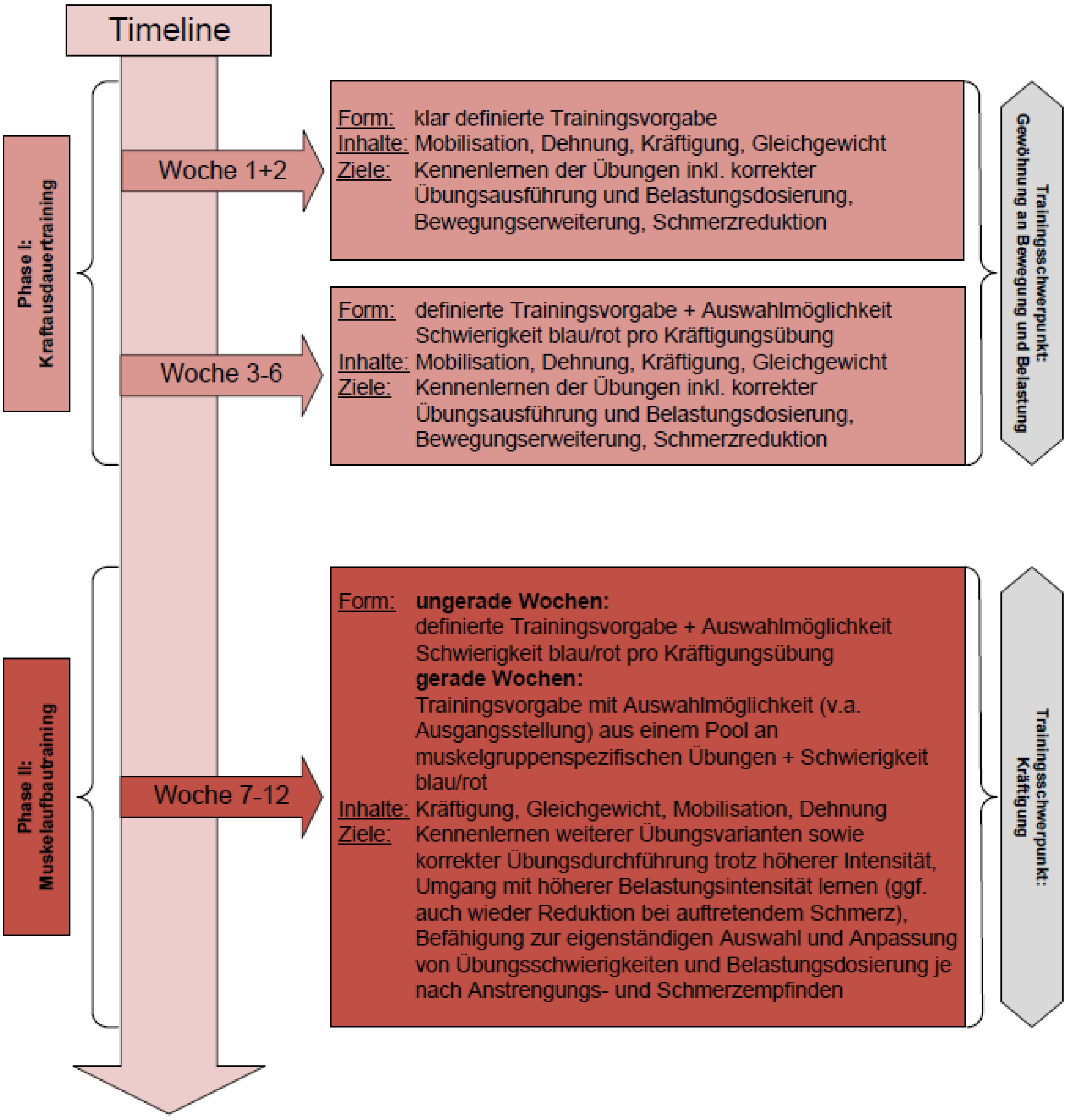


**Abbildung 2: Übersicht Aufbau und Inhalte 12-Wochen-Trainingsintervention für Gonarthrosepatienten.**

## 3.9 Zielmessgrößen

Die folgende Tabelle listet alle Messparameter und die entsprechenden Messinstrumente inklusive Zeitpunkte der Messung auf. Details zu den Zielmessgrößen und Erhebungsinstrumenten finden sich in den folgenden Kapiteln.

| Grunddaten | Beschreibung | Messinstrument (Quelle) | t0a | t0b | t1 | t2 | fortlaufend |
|---|---|---|---|---|---|---|---|
| **Patientencharakteristika** | | | | | | | |
| Teilnehmer Charakteristika | Geburtsdatum, Geschlecht, BMI, Anamnese (relevante Diagnosen und Verletzungshistorie), Abfrage Erfahrung mit Kräftigungsübungen und Technikaffinität | Erhebungsfragebogen in Anlehnung an PAR-Q [12,13], Technikaffinität (TA-EG) [15] | x (teils) | x (teils) | | | |
| VR-12 | Allgemeiner Gesundheitszustand | VR-12 Fragebogen [16] | | x | x | x | |
| KOOS Score | Schmerz, Symptome, Tätigkeiten des Alltags, Funktionsfähigkeit im Sport und in der Freizeit, Lebensqualität im Zusammenhang mit dem betroffenen Knie | KOOS Fragebogen [17] | x (Subskala Schmerz) | x | x | x | |
| TSK-GV | Fear of Movement | Tampa Scale for Kinesiophobia – Deutsche Version (TSK-GV) [18] | | x | x | x | |

| | | | | | | | |
|---|---|---|---|---|---|---|---|
| **Erfassung der körperlichen Leistungsfähigkeit** | | | | | | | |
| Maximalkraftmessung | Isometrische Maximalkraftmessung an den DAVID-Kraftmessgeräten | DAVID-Kraftmessgeräte | | x | x | x | |
| 30s_Chair_Stand_Score | Beinkraft und Beinkraftausdauer über Anzahl durchgeführter Wiederholungen innerhalb 30 Sekunden | 30 Second Chair Stand Test [19] | | x | x | x | |
| Gleichgewicht (COP-Spur oder COP-Geschwindigkeit) | Messung der Gleichgewichtsfähigkeit im Parallelstand, Tandemstand links/rechts, Einbeinstand links/rechts | Zebris Druckmessplatte | | x | x | x | |
| Anstrengung (BORG) | Anstrengungsempfinden RPE 0-10 | BORG-Skala (RPE) 0-10 → Auslesen der LogFiles aus der re.flex App | | | | | *x* *(nach jeder Übung und Trainingseinheit)* |
| **Erfassung der körperlichen und sportlichen Aktivität (alle Gruppen)** | | | | | | | |
| EHIS-PAQ | Bewegungs- und Sportaktivität in einer typischen Woche der letzten 4 Wochen | EHIS-PA Fragebogen [20] | | x | x | x | *zusätzlich nach 4 und 8 Wochen der Interventions-, Warte- oder Follow-up-Phase* |
| Trainingstagebuch | körperliche Aktivität, Schmerz bei körperlicher Aktivität | Paper and pencil | | | | | x (täglich) |

| | | | | | | | | |
|---|---|---|---|---|---|---|---|---|
| **Messgrößen zur Kontrolle und Dokumentation des Trainings mit der App (Gruppe OA und A)** | | | | | | | | |
| Schmerz (im Signalgelenk) | Schmerz im Signalgelenk (Bein mit Orthese/Sensor) NRS 0-10 | NRS-Skala 0-10 → Auslesen der LogFiles aus der re.flex App | | | | | | *x* *(vor und nach der Trainingseinheit, nach jeder Übung* |
| Anzahl absolvierter Trainingseinheiten und Trainingsdauer | zur Beurteilung der Adhärenz | Auslesen der LogFiles aus der re.flex App | | | | | | *x* |
| Bewegungsqualität | Erfassen der Bewegungsqualität, über die Bewegungsrange und Bewegungsgeschwindigkeit der durchgeführten Übungen (mittels 10-Punkte-Skala) | Beschleunigungssensoren der re.flex Technologie | | | | | | *x* |
| **Erfassung der Nutzungsdauer und -art der Orthese (nur Gruppe OA)** | | | | | | | | |
| Tragetagebuch | Tragedauer Orthese (OA), Trageaktivität Orthese (OA) | Paper and pencil | | | | | | x (täglich) |
| **Erleben und Bewerten der App in der Interaktion** | | | | | | | | |
| Nutzerfreundlichkeit | Konstruktion aus validierten Fragebögen zur Erfassung der wahrgenommenen Nutzerfreundlichkeit | - System Usability Scale (SUS) [21,22]<br>- mHealth App Usability Questionnaire (MAUQ) [23]<br>- ergänzende Items von Harder et al. (2017) [24] | | | NWLC | WLC | | |

| | | | | | | | |
|---|---|---|---|---|---|---|---|
| Informationsqualität | Einzelitems zur Erfassung der Informationsqualität | Auswahl der Items [25]:<br>- Appropriate Amount<br>- Concise Representation<br>- Understandability<br>- Relevancy | | | NWLC | WLC | |
| Spezifische Wirkfaktoren einer adäquaten Übungsinstruktion | Erfassung einer adäquaten Übungsinstruktion (Kompetenzerleben, Sicherheitserleben, Bedarfsgemäßheit) und des Feedbacks | Eigenentwurf unter Hinzunahme von 2 Items in Anlehnung an Rackow et al. (2013 [26] und Harder et al. (2017) [24] | | | NWLC | WLC | |
| Patientenzufriedenheit | Erfassung der generellen Zufriedenheit mit der erhaltenen Intervention | ZUF-8 Patientenzufriedenheit [27] | | | NWLC | WLC | |
| **Patientensicherheit** | | | | | | | |
| Minitagebuch mit Adverse Events | Dokumentationsbogen unerwarteter Ereignisse und auftretender Nebenwirkungen während der Interventionszeit | Paper and pencil | | | | | *x*<br>(bei Auftreten unerwarteter Ereignisse) |

x = alle Gruppen, *x = nur Interventionsgruppe OA bzw. A*

### 3.9.1 Patientencharakteristika

Zu t0 werden Alter, Geschlecht, BMI, Anamnese (relevante Diagnosen und vorausgehende Verletzungen und Operationen der UEX und des unteren Rückens), Erfahrung mit Kräftigungsübungen sowie die Technikaffinität erfasst.

### 3.9.2 Patientenberichtete Endpunkte

<u>Knee Osteoarthritis Outcome Score (KOOS) [17]</u>

Fragebogen zur Erfassung von Schmerz, Symptomen, Tätigkeiten des Alltags, der Funktionsfähigkeit im Sport und in der Freizeit sowie der Lebensqualität im Zusammenhang mit dem von Arthrose betroffenen Kniegelenk.

<u>Veterans RAND 12 Item Health Survey (VR-12) [16]</u>

Generischer Fragebogen zur Erfassung der gesundheitsbezogenen Lebensqualität.

<u>Tampa Scale for Kinesiophobia – Deutsche Version (TSK-GV) [18]</u>

Deutsche Version der Tampa Scale zur Erfassung der Bewegungsangst.

<u>Bewegungs- und Sportaktivität (EHIS-PAQ) [20]</u>

Fragebogen zur Erfassung der Bewegungs- und Sportaktivität einer typischen Woche der vergangenen 4 Wochen.

<u>Trainingstagebuch, Orthesen-Tragetagebuch</u>

Während der Interventions- bzw. Warte- und Follow-up-Phase werden täglich Dauer und Art eines körperlichen Trainings bzw. moderater bzw. anstrengender körperlicher Aktivität außerhalb des app-gestützten Trainings dokumentiert (sofern zutreffend). Zudem wird in der Gruppe OA täglich die Tragedauer der Orthese und der Einsatzzweck in der Gruppe OA erfasst. Dies wird zusammengefasst nochmals mit Hilfe des EHIS-PAQ (mit einer Zusatzfrage für die Orthesen-Tragedauer) abgefragt. Dabei werden zusätzlich zu t0, t1 und t2 Zwischenabfragen nach 4 und 8 Wochen der Interventionsphase integriert.

<u>Erleben und Bewerten der Interaktion mit der Software-Applikation</u>

Zur Bewertung der App werden folgende Fragebögen eingesetzt: (1) Nutzerfreundlichkeit mit einer Übersetzung der System Usability Scale nach Brooke (1986) [21], Items des mHealth App Usability Questionnaire (MAUQ) [23] und ergänzende Items von Harder et al. (2017) [24], (2) Informationsqualität (Eigenübersetzung nach Lee et al. (2002) [25], ergänzende Items aus Harder et al. (2017) [24]), (3) spezifische Wirkfaktoren einer adäquaten Übungsinstruktion

(Kompetenzerleben, Sicherheitserleben, Bedarfsgemäßheit) und des Feedbacks (Eigenentwurf unter Hinzunahme von 2 Items in Anlehnung an Rackow et al. (2013) [26] und Harder et al. (2017) [24]) sowie (4) Patientenzufriedenheit mit 8 Items des ZUF-8 [27]. Die Erfassung erfolgt jeweils im Anschluss der app-gestützten 12-wöchigen Intervention.

### 3.9.3 Kraftdiagnostik

DAVID-Kraftmessung

Die Kraftdiagnostik dient der Ermittlung der Maximalkraft sowie der Kraftverhältnisse (Seitenvergleich, Agonist/Antagonist) und agiert als Kenngröße für die Überprüfung der Wirksamkeit der Trainingsintervention. Getestet wird die Maximalkraft der Kniestrecker und -beuger in standardisierten Ausgangsstellungen an DAVID-Kraftgeräten (Schupp GmbH & Co. KG, Dornstetten, F 200 Leg Extension & F 300 Leg Curl). Die Geräte sind CE-zertifiziert und die Anwendung erfolgt lediglich im Rahmen der Zweckbestimmung durch entsprechend geschultes Personal. Der Proband drückt ohne ruckartige Bewegungen mit langsam aufbauendem, maximalem Krafteinsatz isometrisch gegen einen festen Widerstand. Die Probanden werden angehalten nur so viel Kraft aufzubringen, dass keine Schmerzen provoziert werden. Dies wird entsprechend im Case Report Form (CRF) erfasst.

30 Second Chair Stand Test [19]

Zusätzlich zur DAVID-Kraftmessung soll der 30-Second Chair Stand durchgeführt werden, um später gegebenenfalls die Aussagekraft dieses Tests in Bezug auf den weiteren Einsatz als einfaches Diagnostikelement evaluieren zu können. Der 30 Second Chair Stand Test zielt darauf ab, die Beinkraft sowie Beinkraftausdauer zu prüfen. Der Proband sitzt dabei in der Mitte eines Stuhls, die Hände werden verschränkt vor dem Körper gehalten, die Füße setzen plan auf dem Boden auf und der Rücken bleibt gerade. Aufgabe des Probanden ist es, innerhalb von 30 Sekunden so oft wie möglich in eine vollständig aufrechte Körperposition aufzustehen und wieder auf den Stuhl abzusitzen. In Abhängigkeit des Geschlechts und Alters erfolgt eine Einstufung, wie viele Wiederholungen mindestens geschafft werden sollten.

### 3.9.4 Gleichgewichtstest

Die Posturale Stabilität wird mittels einer Druckmessplatte (Zebris GmbH) erfasst. Die Druckmessplatte ist CE-zertifiziert und die Anwendung erfolgt lediglich im Rahmen der Zweckbestimmung durch entsprechend geschultes Personal. Sie zeichnet über Kraftsensoren die Spurlänge des Körperschwerpunkt-Verlaufs (COP-Spurlänge) sowie die COP-Geschwindigkeit auf. Der Gleichgewichtstest auf der Druckmessplatte (stabiler Untergrund) wird im Parallelstand (hüftbreiter, paralleler Stand, Knie leicht gebeugt) mit offenen und geschlossenen Augen, Tandemstand links/rechts (beide Füße direkt hintereinander

aufgestellt, Knie leicht gebeugt, Gewicht gleichmäßig verteilt) und Einbeinstand links/rechts (auf einem Bein stehend, Standbeinknie leicht gebeugt, Schwungbein in der Luft: keine Berührung mit dem Standbein oder Boden) durchgeführt (siehe Abbildung 3). Bei allen Testpositionen ist der Proband in einer aufrechten Position, die Hände sind in den Hüften fixiert und der Blick ist nach vorne gerichtet. Vor jedem Test darf ein Gewöhnungsversuch durchgeführt werden. Gemessen wird die COP-Spurlänge sowie die COP-Geschwindigkeit während je 1 Messung im Parallelstand mit offenen und geschlossenen Augen sowie je 2 Messungen im Tandemstand links/rechts und Einbeinstand links/rechts. Die Messdauer beträgt jeweils 10 s (Parallelstand/Tandemstand) bzw. 6 s (Einbeinstand).

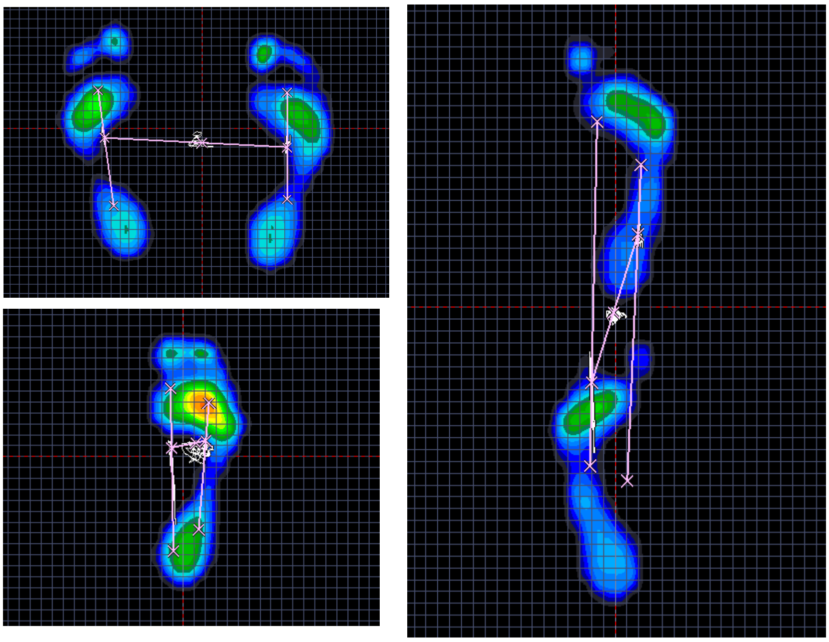

**Abbildung 3: Parallelstand (oben links), Tandemstand rechts (rechts), Einbeinstand rechts (unten links).**

### 3.9.5 Patientensicherheit

Die Probanden erhalten nach Studieneinschluss einen Meldebogen zur Dokumentation aller unerwünschten Ereignisse während der Studiendauer. Im Falle unerwünschter Ereignisse werden diese innerhalb einer Woche an die Studienleitung übermittelt (postalisch, per Email, telefonisch). Unerwünschte Ereignisse, die die Konsultation eines Arztes oder medizinischen Fachpersonals erforderlich machen, müssen der Studienleitung umgehend mitgeteilt werden. Die Einschätzung, ob es sich um eine interventionsbedingte Nebenwirkung handelt, erfolgt durch einen internistischen oder orthopädischen Kollegen der Abt. Sportmedizin. Im Falle einer unklaren Zuordnung oder eines schwerwiegenden Ereignisses werden Vertreter der Fa. Sporlastic und weitere unabhängige Kollegen des UKT in ein Safetyboard berufen. Dieses entscheidet auch über die Weiterführung bzw. den vorzeitigen Abbruch der Studie aus Sicherheitsgründen.

## 3.10 Datenanalyse

Die Daten werden mit den Softwarepaketen IBM SPSS Statistics (SPPS) und R analysiert. Der Vergleich der Patientencharakteristika der Gruppen WLC und NWLC (OA und A) zu t0 erfolgt deskriptiv sowie in Abhängigkeit des Skalenniveaus und der Verteilung mit parametrischen bzw. nicht-parametrischen Testverfahren. Zur Überprüfung der Interventionseffekte wird eine 2x2x2 mixed ANOVA mit den Zwischensubjekt-Faktoren *Warteliste* (WLC, NWLC) und *Intervention* (OA, A) und dem Innersubjekt-Faktor *Zeit* (t0, t1) durchgeführt. Der Gesamtinterventionseffekt wird hierbei durch die Interaktion *Warteliste x Zeit* abgebildet. Der Vergleich der Interventionsformen OA und A erfolgt ergänzend mit einer 2x2 mixed ANOVA mit den Zwischensubjekt-Faktor *Intervention* (OA, A) und dem Innersubjekt-Faktor *Zeit* (t0, t1). Dieser Vergleich berücksichtigt nur Probanden der NWLC. Alle Varianzanalysen werden durch Kalkulation des *generalized eta squared* ($\eta^2_G$) ergänzt, um die Varianzaufklärung der einzelnen Faktoren bzw. deren Interaktion zu berechnen.

Der ergänzenden Effektstärkenberechnung werden alle gültigen Datensätze zugrunde gelegt, also auch die post-Intervention-Werte der WLC. Unter der Annahme, dass die Zeit keinen substantiellen Effekt auf die abhängige Variable hat, werden der Effektstärkenberechnung folgende Daten zugrunde gelegt: für NWLC (OA und A) t0 (Pre) und t1 (Post), für WLC t0 (Pre1) und t2 (Post). Eine ergänzende Effektstärkenberechnung erfolgt für NWLC für den Zeitraum t0 (Pre) und t2 (FU). Die Effektstärken und 95% Konfidenzintervalle der mittleren Differenzen zwischen den genannten Zeitpunkten werden mit gängigen Verfahren unter Berücksichtigung der Verteilung der mittleren Differenzen und der Stichprobengröße berechnet [28].

Explorative Subanalysen werden unter Berücksichtigung relevanter Einflussvariablen wie beispielsweise Alter, Schwergrad der Erkrankung, technische Affinität und sportliche Vorerfahrung durchgeführt.

# 4 Zeitplanung

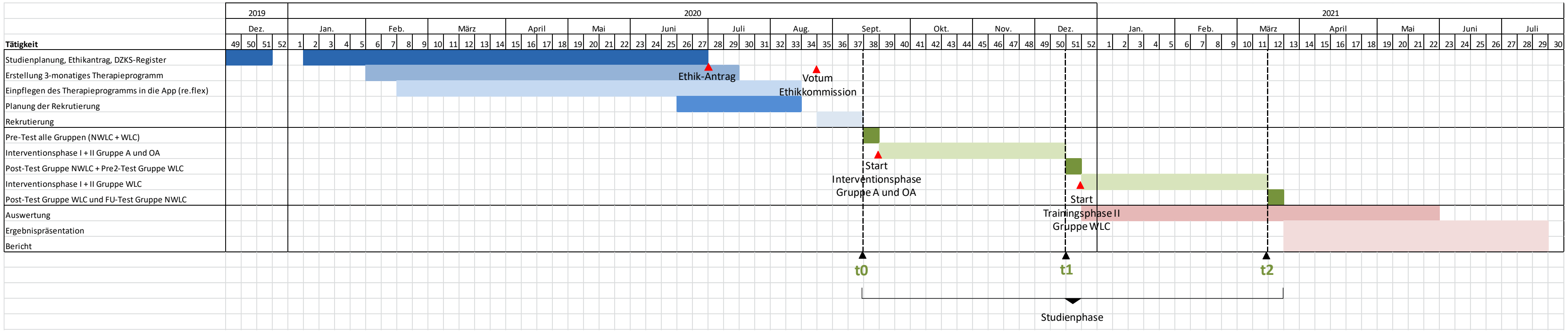

# 5 Datenschutz

## 5.1 Probandenkennung und Datenbank

Sämtliche in der Studie erfassten Daten werden ausschließlich unter einem vom Studienleiter festgelegten Pseudonym gespeichert. Dieses wird nach dem endgültigen Studieneinschluss an die Teilnehmer anhand einer Probandenidentifikationsliste vergeben. Eine Rückführung der Daten zu den Personendaten ist nur durch Einsicht der Probanden-Reidentifikationsliste möglich. Der Zugriff auf diese Datei ist nur den auf Seite 1 des Prüfplans genannten Mitarbeitern der Studie und dem Systemadministrator möglich.

Die Dauer der Aufbewahrung der Studienunterlagen richtet sich nach den aktuellen gesetzlichen Vorgaben.

Alle digitalen sowie auf Erhebungsbögen erfassten Daten werden nach Abschluss der Studie mindestens 10 Jahre elektronisch gespeichert. Auf Anfrage der Probanden kann eine vorzeitige Löschung der Daten vorgenommen werden. Die Re-Identifikation erfolgt in diesem Fall durch die oben genannte Liste. Bis dato erfolgte Auswertungen und Publikationen bleiben von der Löschung unberührt.

## 5.2 Wahrung der ärztlichen Schweigepflicht

Alle an der Studie beteiligten Mitarbeiter unterliegen der ärztlichen Schweigepflicht.

# 6 Versicherungsschutz

Es wird ein Versicherungsschutz für die An- und Abreise zu den Messungen der Studie (Wege-Unfall-Versicherung) in der Abteilung Sportmedizin des UKT abgeschlossen. Eine gesonderte Probandenversicherung für die Teilnehmer wird nicht abgeschlossen. Die Probandeninformation enthält einen entsprechenden Hinweis. Sämtliche unerwartete Ereignisse und auftretende Nebenwirkungen während der Interventionszeit werden in einem Minitagebuch „Adverse Events“ dokumentiert.

# 7 Vor- und Nachteile für Probanden / Risiko

Im folgenden Abschnitt sollen die für den Teilnehmer möglichen Vorteile, Nachteile und Risiken dargestellt werden. Die Probanden erhalten keine Vergütung für die Teilnahme.

Vorteile:

Während der Studie wird die körperliche Leistungsfähigkeit der Probanden über standardisierte Verfahren gemessen. Jeder Teilnehmer erhält über 12 Wochen ein systematisches digitales Trainingsprogramm inklusive Sensortechnik (+ ggf. Orthese). Das Trainingsprogramm erfordert keine Präsens in der Sportmedizin Tübingen und kann komplett zu Hause absolviert werden. Jeder Studienteilnehmer erhält ein Set an Minibändern der Firma Sporlastic.

Nachteile:

Nachteile durch die Teilnahme an der Studie sind in dem hohen zeitlichen Aufwand zu sehen. Für die Messzeitpunkte (t0, t1 und t2) wird von 2 – 2,5 h pro Teilnehmer und Messtermin ausgegangen. Zudem sollen die Probanden das Training 3-4x wöchentlich durchführen.

Risiken:

Mit der Teilnahme an der Studie sind keine besonderen Risiken verbunden, da es sich bei den vorgestellten Untersuchungsverfahren um nicht-invasive Methoden handelt. Die Sporttauglichkeit wird vorab durch einen Fragebogen und ggf. einer ärztlichen Untersuchung ermittelt. Zudem erfolgt die Indikation und Tragemodus für die Orthese (GENUDYN® OA, Firma Sporlastic) im Rahmen der ärztlichen Eingangsuntersuchung.

Druckerscheinungen von Haut und Nerven sowie Zirkulationsbeeinträchtigungen können bei sachgemäßer Anpassung der Orthese sowie der Sensoren weitestgehend ausgeschlossen werden.

Sollten während dem Training mit dem re.flex System zu irgendeinem Zeitpunkt ungewöhnliche Beschwerden, Müdigkeit oder sogar Schmerzen verspürt werden, wird der Proband angehalten das Training zu unterbrechen und das unerwünschte Ereignis der Studienleitung mitzuteilen und abzuklären, bevor das Training wieder aufgenommen wird.

# 8 Interessenskonflikt

Das Studienvorhaben wird in Kooperation mit der Firma Sporlastic GmbH durchgeführt und finanziert.

# Literaturverzeichnis

[1] Fuchs J, Kuhnert R, Scheidt-Nave C (2017) 12-Monats-Prävalenz von Arthrose in Deutschland. Journal of Health Monitoring 2(3):55–60. DOI 10.17886/RKI-GBE-2017-054

[2] AWMF (2018). S2k-Leitlinie Gonarthrose. Zugriff am 14.April.2020 unter https://www.awmf.org/uploads/tx_szleitlinien/033-004l_S2k_Gonarthrose_2018-01_1.pdf

[3] Krauß, I. (2016). Sport-und Bewegungstherapie bei Gon-und Coxarthrose. *Dtsch Z Sportmed*, *67*, 276-281.

[4] Fransen, M., McConnell, S., Harmer, A. R., Van der Esch, M., Simic, M., & Bennell, K. L. (2015). Exercise for osteoarthritis of the knee. *Cochrane Database of Systematic Reviews*, (1).

[5] Brosseau, L., Taki, J., Desjardins, B., Thevenot, O., Fransen, M., Wells, G. A., ... & Gifford, W. (2017). The Ottawa panel clinical practice guidelines for the management of knee osteoarthritis. Part two: strengthening exercise programs. *Clinical rehabilitation*, *31*(5), 596-611.

[6] Bossen, D., Veenhof, C., Dekker, J., & de Bakker, D. (2013). The usability and preliminary effectiveness of a web-based physical activity intervention in patients with knee and/or hip osteoarthritis. *BMC medical informatics and decision making*, *13*(1), 61.

[7] Bossen, D., Veenhof, C., Van Beek, K. E., Spreeuwenberg, P. M., Dekker, J., & De Bakker, D. H. (2013). Effectiveness of a web-based physical activity intervention in patients with knee and/or hip osteoarthritis: randomized controlled trial. *Journal of medical Internet research*, *15*(11), e257.

[8] Schwarze, M., Bartsch, L. P., Block, J., Wolf, S. I., & Alimusaj, M. (2019). Einlagen, Knie-und Unterschenkelorthesen in der Behandlung der medialen Gonarthrose. *Der Orthopäde*, 1-11.

[9] Moyer, R. F., Birmingham, T. B., Bryant, D. M., Giffin, J. R., Marriott, K. A., & Leitch, K. M. (2015). Valgus bracing for knee osteoarthritis: a meta-analysis of randomized trials. *Arthritis care & research*, *67*(4), 493-501.

[10] Bundesinstitut für Arzneimittel und Medizinprodukte (2020). Das Fast-Track-Verfahren für digitale Gesundheitsanwendungen (DiGA) nach § 139e SGB V. Ein Leitfaden für Hersteller, Leistungserbringer und Anwender. Zugriff am 27.04.2020 unter https://www.bfarm.de/SharedDocs/Downloads/DE/Service/Beratungsverfahren/DiGA-Leitfaden.pdf;jsessionid=198BB7AC0C6E3149F07A563359369D1C.1_cid323?__blob=publicationFile&v=1

[11] Fuchs, J., Kuhnert, R., & Scheidt-Nave, C. (2017). 12-Monats-Prävalenz von Arthrose in Deutschland.

[12] Canadian Society for Exercise Physiology (2012). Physical Activity Readiness Questionnaire – PAR-Q. Zugriff am 05.05.2020 unter http://westpointgrey.org/wp-content/uploads/2014/02/PAR-QXXForm.pdf.

[13] Deutsche Gesellschaft für Sportmedizin und Prävention (DGSP) (2012). PAR-Q Fragebogen (deutsche Fassung). Zugriff am 05.05.2020 unter https://daten2.verwaltungsportal.de/dateien/seitengenerator/leitlinie_vorsorgeuntersuchung_4.10.2007-anlage-1.pdf.

[14] Altman, R., Asch, E., Bloch, D., Bole, G., Borenstein, D., Brandt, K., ... & Howell, D. (1986). Development of criteria for the classification and reporting of osteoarthritis: classification of osteoarthritis of the knee. *Arthritis & Rheumatism: Official Journal of the American College of Rheumatology*, *29*(8), 1039-1049.

[15] Karrer, K., Glaser, C., Clemens, C., & Bruder, C. (2009). Technikaffinität erfassen–der Fragebogen TA-EG. *Der Mensch im Mittelpunkt technischer Systeme*, *8*, 196-201.

[16] Iqbal, S. U., Rogers, W., Selim, A., Qian, S., Lee, A., Ren, X. S., & Kazis, L. (2007). The Veterans RAND 12 Item Health Survey (VR-12): what it is and how it is used. *CHQOERs VA Medical Center. Bedford, MA: CAPP Boston University School of Public Health*, 1-12.

[17] Kessler, S., Lang, S., Puhl, W., & Stöve, J. (2003). Der knee injury and osteoarthritis outcome score-ein funktionsfragebogen zur outcome-messung in der knieendoprothetik. *Zeitschrift für Orthopädie und ihre Grenzgebiete*, *141*(03), 277-282.

[18] Rusu, A. C., Kreddig, N., Hallner, D., Hülsebusch, J., & Hasenbring, M. I. (2014). Fear of movement/(Re) injury in low back pain: confirmatory validation of a German version of the Tampa Scale for Kinesiophobia. *BMC musculoskeletal disorders*, *15*(1), 280.

[19] Centers for Disease Control and Prevention (2017). Assessment 30-Second Chair Stand. Zugriff am 06.05.2020 unter https://www.cdc.gov/steadi/pdf/STEADI-Assessment-30Sec-508.pdf.

[20] Finger, J. D., Tafforeau, J., Gisle, L., Oja, L., Ziese, T., Thelen, J., ... & Lange, C. (2015). Development of the European health interview survey-physical activity questionnaire (EHIS-PAQ) to monitor physical activity in the European Union. *Archives of Public Health*, *73*(1), 1-11.

[21] Brooke, J. (1996). SUS-A quick and dirty usability scale. *Usability evaluation in industry*, *189*(194), 4-7.

[22] Albert, W., & Tullis, T. (2013). *Measuring the user experience: collecting, analyzing, and presenting usability metrics*. Newnes.

[23] Zhou, L., Bao, J., Setiawan, I. M. A., Saptono, A., & Parmanto, B. (2019). The mHealth APP usability questionnaire (MAUQ): development and validation study. *JMIR mHealth and uHealth*, *7*(4), e11500.

[24] Harder, H., Holroyd, P., Burkinshaw, L., Watten, P., Zammit, C., Harris, P. R., ... & Jenkins, V. (2017). A user-centred approach to developing bWell, a mobile app for arm and shoulder exercises after breast cancer treatment. *Journal of Cancer Survivorship*, *11*(6), 732-742.

[25] Lee, Y. W., Strong, D. M., Kahn, B. K., & Wang, R. Y. (2002). AIMQ: a methodology for information quality assessment. *Information & management*, *40*(2), 133-146.

[26] Rackow, P., Scholz, U., & Hornung, R. (2013). The German psychological need satisfaction in exercise scale. *Swiss Journal of Psychology*.

[27] Schmidt, J., Wittmann, W. W., med Psychol, V. P., & CSQ, C. S. Q. (2002). Fragebogen zur Messung der Patientenzufriedenheit. In *Diagnostische verfahren in der psychotherapie. Göttingen: Hogrefe* (pp. 392-6).

[28] Lee, D. K. (2016). Alternatives to P value: confidence interval and effect size. *Korean journal of anesthesiology*, *69*(6), 555.

## Anlagen

Anlage 1: Probandeninformation

Universitätsklinikum Tübingen

### Probandeninformation zur Studie mit dem Titel:

Randomisiert-kontrollierte Studie im Wartelisten-Kontroll-Design zur Überprüfung einer 12-wöchigen app- und orthesengestützten Trainingsintervention bei Patienten mit mittel- bis schwergradiger Gonarthrose

Sehr geehrte Teilnehmerin, sehr geehrter Teilnehmer[4],

die Abteilung der Sportmedizin des Universitätsklinikum Tübingen führt in Kooperation mit der Firma Sporlastic GmbH eine Studie zur Überprüfung einer 12-wöchigen app- und orthesengestützten Trainingsintervention bei Patienten mit mittel- bis schwergradiger medialer oder lateraler Kniearthrose durch.

Im Folgenden möchten wir Sie mit dem Ablauf der Studie bekannt machen und die Inhalte genauer erläutern. Alle Fragen, die nach mündlicher und schriftlicher Information nicht geklärt sind, können Sie jederzeit an die oben genannten Ansprechpartner richten.

### 1. Worum geht es in dieser Studie?

Arthrose gilt weltweit als die am häufigsten vorkommende degenerative Gelenkerkrankung. Mehr als die Hälfte der Betroffenen in Deutschland leiden dabei an einer Gonarthrose, also einem Befall des Kniegelenks. Mit zunehmendem Schweregrad der Gonarthrose werden häufiger von Schmerzen, Einschränkungen der körperlichen Funktionsfähigkeit sowie einer verminderten Lebensqualität seitens der Patienten berichtet. Als konservative Therapieform wird von den nationalen und internationalen Leitlinien die Sport- und Bewegungstherapie empfohlen. Bei entsprechend trainingswirksamer Belastungsdosierung zeigten verschiedene Trainingsformen zur Muskelkräftigung sowie Verbesserung der Gleichgewichtsfähigkeit und Beweglichkeit einen kurz- und mittelfristigen positiven Behandlungseffekt. Dennoch gibt es weiter Bedarf an der Entwicklung eines Programms, das zur langfristigen Erhaltung eines regelmäßig durchgeführten Übungsprogramms motiviert.

[4] Im Folgenden erfolgt aus Gründen der Übersichtlichkeit die Anrede ausschließlich in der männlichen Form, alle Geschlechter sind damit gleichermaßen angesprochen.

Des Weiteren konnte auch der Einsatz orthopädischer Hilfsmittel, beispielsweise in Form von Knieorthesen, bereits positive Wirksamkeitsnachweise zeigen. Hier ist zu prüfen, ob bei einem ergänzenden Einsatz zur Sport- und Bewegungstherapie möglicherweise sogar zusätzliche Effekte erzielt und die körperliche Aktivität weiter gesteigert werden kann.

Hauptziel der Studie ist die Untersuchung der Wirksamkeit und Unbedenklichkeit einer 12-wöchigen app-gestützten Trainingsintervention im Vergleich zu einer Kontrollgruppe ohne Intervention. Die Wirksamkeit soll sowohl für die alleinige Nutzung einer App als auch in Kombination mit einer Knieorthese überprüft werden. Die Trainingsintervention beinhaltet dabei ein Programm aus Übungen zur Kräftigung, Beweglichkeit, Mobilisation und Dehnfähigkeit. Die Studie wird mit 60 Probanden im Warte-Kontroll-Design durchgeführt, so dass im Verlauf alle Studienteilnehmer eine Trainingsintervention mit der App erhalten, die Hälfte zusätzlich eine Knieorthese. Die Studie wird in Kooperation mit der Firma Sporlastic GmbH durchgeführt und von dieser finanziert.

## 2. Wie ist der Ablauf der Studie und was muss ich bei der Teilnahme beachten?

Als Voraussetzung zur Studienteilnahme werden zunächst einige Informationen zu Ihrer Person, Ihrem aktuellen Gesundheitszustand und Ihrer körperlichen Leistungsfähigkeit ermittelt. Ein Teil dieser Informationen wird bereits bei der ersten Kontaktaufnahme am Telefon oder via Email erfasst. Die Studiendauer für den einzelnen Probanden beträgt 27 Wochen. Der erste Vor-Ort-Termin in der Sportmedizin Tübingen, beinhaltet zunächst ein Informationsgespräch. Nach erfolgter Einwilligung zur Studie findet eine ärztliche Eingangsuntersuchung (t0) zur Überprüfung der Diagnose der Kniearthrose sowie die Testung der körperlichen Leistungsfähigkeit inklusive Kraftdiagnostik, Gleichgewichtstest und einiger studienrelevanter Fragebögen statt (Details weiter unten). Anschließend werden Sie per Zufall in die Therapiegruppe (NWLC) oder die Warteliste (WLC) zugeteilt. Gehören Sie der Therapiegruppe an, so werden Sie erneut per Zufall in die Gruppe „app-gestütztes Training“ (A) oder orthesen- und appgestütztes Training“ (OA) zugeteilt. Es folgt die 12-wöchige Therapie- bzw. Wartephase (Details hierzu weiter unten). Danach erfolgen erneut eine Messung (t1) und die zufällige Zuteilung der Probanden der Wartelistengruppe (WLC) in eine der beiden Interventionsgruppen für die folgenden 12 Wochen. Die beiden Gruppen, die bereits in der ersten Phase die Intervention erhalten haben, bekommen für die folgenden 12 Wochen keine Intervention mehr. Nach dieser zweiten Interventions- bzw. Wartephase erfolgt eine weitere Messung (t2).

Die folgende Grafik (Abbildung 1) gibt Ihnen einen Überblick über den Studienablauf.

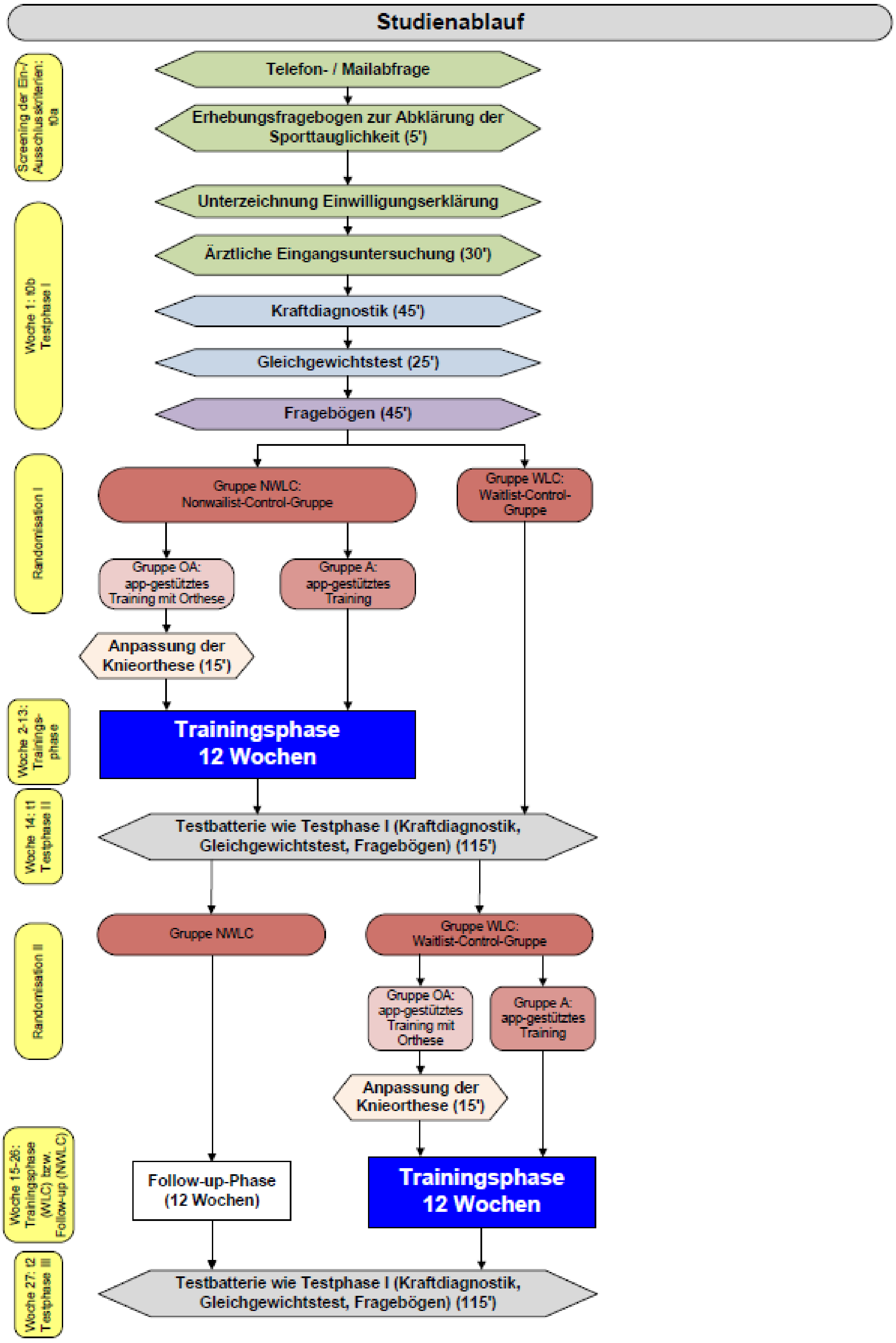


**Abbildung 4: Studienablauf**

## 3. Studienbedingte Untersuchungen

Wie oben beschrieben sind insgesamt 3 Untersuchungstermine (t0, t1 und t2) zur Durchführung der Testbatterie (ca. 145 min) vorgesehen. Die Testbatterie umfasst die folgenden Inhalte:

**Ärztliche Eingangsuntersuchung**
Die orthopädische Eingangsuntersuchung dient der Diagnosesicherung einer medialen oder lateralen Kniearthrose, der Abklärung möglicher Kontraindikationen bezüglich des Tragens einer Orthese bzw. der Durchführung des Trainingsprogramms sowie der Orthesenauswahl und des Tragemodus (Valgus-/Varus-Orthese).

**Kraftdiagnostik**
Die Kraftdiagnostik dient der Ermittlung Ihrer aktuellen Kraftverhältnisse der Oberschenkelmuskulatur. Getestet wird Ihre Maximalkraft der Kniestrecker und –beuger in standardisierter Ausgangsstellung an Geräten der Firma DAVID. Zudem wird die Beinkraft und Beinkraftausdauer zusätzlich über den 30 Second Chair Stand Test beurteilt.

**Gleichgewichtstest**
Ihre Gleichgewichtsfähigkeit wird im Parallelstand, Tandemstand und Einbeinstand ermittelt. Die Testung erfolgt mit einer Druckmessplatte der Firma Zebris GmbH.

**Fragebögen**
Vor (t0), unmittelbar nach dem Training (t1) sowie 12 Wochen nach der Intervention (t2) (Gruppe NWLC) bzw. zu Studienbeginn (t0), nach Ende des Kontrollzeitraums (t1) sowie 12 Wochen nach Trainingsbeginn (t2) (Gruppe WLC) bitten wir Sie Fragebögen (Dauer ca. 45 Min.) zu den folgenden Inhalten auszufüllen:

- Allgemeine Fragen zu Ihrer Person
- Fragen zu kniearthrosespezifischen Zusammenhängen
- Fragen zu Ihrer gesundheitsbezogenen Lebensqualität
- Fragen zur Bewegungsangst
- Fragen zur Bewegungs- und Sportaktivität (wird zusätzlich alle 4 Wochen abgefragt)
- Fragen zum Erleben und Bewerten der Interaktion mit der App (nur einmalig nach dem 12-wöchigen Interventionsprogramm).

Während der Interventionsphase werden Sie zudem bei jeder Trainingseinheit Angaben zum **Anstrengungs- und Schmerzempfinden** machen. Zudem sollen Sie in einem **Minitagebuch** eintragen, ob unerwartete Ereignisse bzw. Nebenwirkungen während der Interventionszeit hervorgerufen werden und welche zusätzlichen Aktivitäten Sie über den Tag durchgeführt haben.

## 4. Wie gestaltet sich das Trainingsprogramm?

Die Trainingsintervention erstreckt sich über 12 Wochen und beinhaltet zwei Phasen mit jeweils 6 Wochen. Im Vordergrund steht die Kräftigung der Muskelgruppen Kniestrecker, Kniebeuger und Hüftabspreizer sowie Übungen zur Mobilisation, Dehnung und Gleichgewichtsfähigkeit. Phase I ist gekennzeichnet durch eine allmähliche Heranfürhung an die Trainingsbelastung und einem Kraftausdauertraining

mit eher geringen Belastungsintensitäten. Phase II wird als Muskelaufbautraining durchgeführt, das Training wird also etwas intensiver. Je nach Gruppenzuteilung wird die Trainingsintervention als app-gestütztes Training oder als app-gestütztes Training in Kombination mit einer Knieorthese durchgeführt. Dazu werden Beschleunigungssensoren jeweils an dem von der Kniearthrose betroffenen Bein befestigt. Die App fungiert dabei als Trainingspartner, der die Übungen inklusive Übungsbeschreibungen und –videos, Wiederholungszahlen, Anzahl an Durchgängen, Bewegungsumfang der Übung sowie die Bewegungsgeschwindigkeit vorgibt. Anfangs geht es zunächst darum, Übungen kennenzulernen und korrekt auszuführen sowie den Umgang mit einer optimalen Belastungsdosierung zu erlernen. Im Verlauf sollen Sie dazu befähigt werden eigenständige Anpassungen der Übungsschwierigkeit je nach individuellem Anstrengungs- und Schmerzempfinden vorzunehmen. Insgesamt sind 3 Trainingseinheiten mit einer Dauer von jeweils 20-25 min pro Übungswoche geplant. Um eine zunehmende Steigerung des Therapieprogramms zu erreichen, werden neben verschiedenen Übungen und Übungsvarianten (z.B. im kurzen oder langen Hebel) auch Minibänder zur Verstärkung des Widerstandes als Trainingsmittel zum Einsatz kommen.

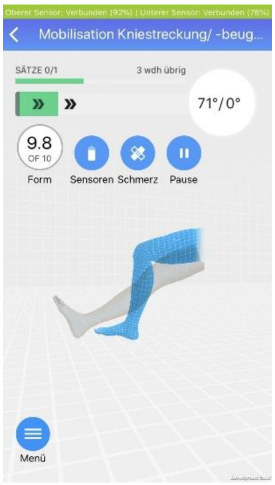


**Abbildung 5: Beispielübung aus der re.flex App**

## 5. Bringt mir die Teilnahme persönliche Vorteile?

Während der Studie wird Ihre körperliche Leistungsfähigkeit über standardisierte Verfahren gemessen. Dabei erhalten Sie über 12 Wochen ein systematisches digitales Trainingsprogramm inklusive Sensortechnik (+ggf. Orthese). Das Trainingsprogramm erfordert keine Präsens und kann komplett zu Hause absolviert werden.

## 6. Welche möglichen Risiken und Belastungen sind zu beachten?

Um das Risiko von unerwarteten Zwischenfällen zu minimieren, wird vor Beginn der Studie Ihre Sporttauglichkeit abgefragt. Zudem wird die aktuelle körperliche Leistungsfähigkeit ermittelt. Die Indikation und Tragemodus für die Orthese erfolgt im

Rahmen der ärztlichen Eingangsuntersuchung. Somit versuchen wir Ihre Belastbarkeit und Tauglichkeit für das Trainingsprogramm bereits vorab festzustellen.

**Kraftmessung**:
Da bei der Messung ein maximaler Krafteinsatz erforderlich ist, kann es zu vorübergehenden muskulären Beschwerden kommen. Des Weiteren ist bei dieser Messung darauf zu achten eine Pressatmung zu vermeiden, da hieraus Kreislaufbeschwerden entstehen können. Sie werden jedoch vor und während des Tests nochmals auf eine gleichmäßige Atmung hingewiesen, um dieses Risiko zu minimieren.

**Gleichgewichtstest:**
Die Messungen der Gleichgewichtsfähigkeit finden auf festem und stabilem Untergrund statt, sodass die Gefahr eines Sturzes als sehr gering erachtet wird. Lediglich bei den Messungen der Gleichgewichtsfähigkeit im Tandemstand und im Einbeinstand, also Positionen mit geringerer Unterstützungsfläche, scheinen Stürze möglich. Dem wird jedoch durch Haltemöglichkeiten in Greifweite des Probanden entgegengewirkt.

**Training:**
Sporttherapeutische Interventionsmaßnahmen haben grundsätzlich ein sehr geringes Behandlungsrisiko. In vereinzelten Fällen kann es durch die Therapiemaßnahme zu einer kurzfristigen Verstärkung der Beschwerdesymptomatik kommen. Das Training kann insbesondere zu Beginn zu muskulären Schmerzen führen (Muskelkater). Hierbei handelt es sich jedoch um einen natürlichen und unschädlichen Anpassungsvorgang an die Belastung. Weitere seltene, aber dennoch mögliche unerwünschte Nebenwirkungen sind Stürze mit damit einhergehenden möglichen Folgeschäden, sowie kardio-vaskuläre Beschwerden, sofern eine Begleiterkrankung des Herz-Kreislauf-Systems vorliegt.

**Orthesen- und Sensortechnologie:**
Die eingesetzte Orthese und die Sensortechnologie besitzen jeweils eine CE-Kennzeichnung als zugelassenes Medizinprodukt. Die Indikationsstellung zum Tragen einer Orthese wird vorab im Rahmen der ärztlichen Eingangsuntersuchung gestellt. Druckerscheinungen von Haut und Nerven sowie Zirkulationsbeeinträchtigungen können bei sachgemäßer Anpassung der Orthese (wird durch einen ausgebildeten Orthopädietechniker angepasst) und Sensoren weitestgehend ausgeschlossen werden.

**Dauer:**
Neben den genannten Risiken ist die zeitliche Dauer sowohl der Untersuchungen (3x ca. 145 Minuten) als auch des Trainings (3x/Woche à 20-25 Minuten) als Belastung zu berücksichtigen.

## 7. Wer darf an dieser Studie nicht teilnehmen?

In die Studie werden Patienten mit mittleren bis schweren Beschwerden aufgrund einer Kniearthrose eingeschlossen. Dabei ist eine uneingeschränkte körperliche Tauglichkeit für die Durchführung des Trainings Voraussetzung. Bereits am Kniegelenk mit einem künstlichen Gelenk versorgte Personen bzw. Personen mit einer das gesamte Kniegelenk (innen und außen) betreffenden Arthrose sowie schwerwiegenden anderen Erkrankungen oder Beschwerden können nicht eingeschlossen werden. Die abschließende Überprüfung dieser Ein- und Ausschlusskriterien erfolgt im Rahmen der ärztlichen Eingangsuntersuchung.

## 8. Wie wird mit meinen persönlichen Daten umgegangen?

Die Durchführung des Forschungsvorhabens erfordert es, dass von Ihnen personenbezogene Daten, insbesondere Angaben über Ihre Gesundheit, körperliche Leistungsfähigkeit sowie Sport- und Bewegungsaktivitäten erhoben, aufgezeichnet und verarbeitet werden. Die erhobenen Daten werden für die wissenschaftliche Auswertung und Überwachung des Forschungsvorhabens sowie für die Archivierung der Ergebnisse verwendet. Die Erhebung, Verarbeitung, Weitergabe und Speicherung der Daten unterliegt strengen gesetzlichen Bestimmungen, die restriktiv eingehalten werden. Nähere Informationen erhalten Sie in der Einwilligungserklärung unter dem Punkt: Datenschutz.

## 9. Besteht ein Versicherungsschutz?

Ein gesonderter Versicherungsschutz besteht für die An- und Abreise zu den Messungen der Studie in der Abt. Sportmedizin der Universitätsklinik Tübingen (Wege-Unfall-Versicherung). Versichert sind Unfälle die sich auf dem direkten Weg vom jeweiligen Wohnort zum Untersuchungsort oder auf dem Rückweg ereignen. Der Versicherungsschutz entfällt, wenn die normale Dauer des Weges verlängert bzw. der Weg selbst durch rein private Maßnahmen unterbrochen wird. Die Ersatzleistung bei Invalidität beträgt 100.000,00 €, bei Tod 50.000,00 €. Versicherungsträger ist die SV Sparkassen Versicherungs-AG Hessen-Nassau-Thüringen. Es wird keine gesonderte Probandenversicherung abgeschlossen, welche Unfälle und Schäden während der Intervention und Durchführung der Diagnostik abdeckt.

Bitte beachten Sie, dass Sie bei Auftreten einer unerwarteten Gesundheitsbeeinträchtigung – unabhängig davon, ob Sie mit der Studie in Zusammenhang steht oder nicht, angehalten sind, diese zunächst der die Studie durchführenden Einrichtung, in diesem Fall der Abteilung Sportmedizin am Universitätsklinikum Tübingen, unverzüglich zu melden. In einem solchen Fall sind Sie zudem angehalten, zweckmäßige Maßnahmen zu treffen, die der Aufklärung der Ursache und des Umfangs des eingetretenen Schadens und der Minderung dieses Schadens dienen.

## 10. Kann ich meine Teilnahme jederzeit beenden?

Wenn Sie aus dem Forschungsvorhaben ausscheiden möchten, können Sie ihre Einwilligung jederzeit und ohne Angaben von Gründen wiederrufen. Durch den Widerruf entstehen Ihnen keinerlei Nachteile.

Unter gewissen Umständen ist es auch möglich, dass der Prüfarzt oder der für die Studie Verantwortliche entscheidet, Ihre Teilnahme an der wissenschaftlichen Prüfung vorzeitig zu beenden, ohne dass Sie auf die Entscheidung Einfluss haben. Die Gründe hierfür können z.B. sein:

- Eine weitere Teilnahme ist aus ärztlicher Sicht nicht mehr vertretbar.
- Es gibt relevante Abweichungen vom Studienprotokoll, die nach Einschätzung der Prüfärzte bzw. der Verantwortlichen zu einer Verfälschung der Studie führen können.

Anlage 2: Einverständniserklärung

## Einverständniserklärung für Probanden

### Titel der Studie

**Randomisiert-kontrollierte Studie im Wartelisten-Kontroll-Design zur Überprüfung einer 12-wöchigen app- und orthesengestützten Trainingsintervention bei Patienten mit mittel- bis schwergradiger Gonarthrose**

- ➔ Bitte lesen Sie dieses Formular sorgfältig durch.
- ➔ Bitte fragen Sie den Untersucher oder Ihre Kontaktperson, wenn Sie etwas nicht verstehen oder wenn Sie mehr wissen möchten.

Ort des Studienzentrums:

UntersucherIn: ______________________________

ProbandIn Vorname und Name: ______________________________

Geburtsdatum: ______________________________

Adresse/ PLZ/ Wohnort: ______________________________

______________________________

______________________________

Telefon/ E-Mail: ______________________________

______________________________

- ➔ Ich nehme an dieser Studie freiwillig teil und kann jederzeit ohne Angaben von Gründen meine Zustimmung zur Teilnahme wiederrufen, ohne dass mir deswegen Nachteile entstehen.
- ➔ Ich wurde mündlich und schriftlich über die Ziele, den Ablauf der Studie, über mögliche Vor- und Nachteile sowie über eventuelle Risiken informiert.
- ➔ Ich habe die zur oben genannten Studie vorliegende schriftliche Probandeninformation gelesen. Meine Fragen im Zusammenhang mit der Teilnahme an dieser Studie sind mir zufriedenstellend beantwortet worden. Ich kann die schriftliche Probandeninformation behalten und erhalte auf Wunsch eine Kopie meiner schriftlichen Einverständniserklärung.
- ➔ Ich hatte genügend Zeit, um meine Entscheidung zu treffen.
- ➔ Ich bin mir bewusst, dass während der Studie die in der Probandeninformation genannten Anforderungen und Vorgaben einzuhalten sind. Im Interesse meiner Gesundheit kann mich die untersuchende Person auch ohne gegenseitiges Einverständnis von der Studie ausschließen oder zum Abbruch der Teilnahme zwingen.

- ➔ Zudem informiere ich den Studienleiter/die Studienleiterin über eine Erkrankung oder ärztliche Behandlung sowie über die Einnahme von Medikamenten (vom Arzt/der Ärztin verordnete oder selbstständig gekaufte).
- ➔ Ich wurde über den Umgang mit personenbezogenen Daten informiert:

## Einwilligungserklärung zum Umgang mit den in einer Studie erhobenen Daten:

- ➔ Ich erkläre, dass ich mit der im Rahmen der Studie erfolgenden Erhebung und Verarbeitung von Daten und ihrer verschlüsselten (pseudonymisierten) Weitergabe einverstanden bin.
- ➔ Die Forschungsergebnisse aus der Studie können in anonymisierter Form in Fachzeitschriften oder in wissenschaftlichen Datenbanken veröffentlicht werden. Bei der Veröffentlichung der Forschungsergebnisse wird meine Identität nicht bekannt. Die Studienleitung vor Ort kann jedoch mit Hilfe einer Patientenliste bei Rückfragen die Daten zu Ihrer Person zurückführen.
- ➔ Ich wurde darüber informiert, dass ich jederzeit Auskunft über meine gespeicherten Daten und die Berichtigung von fehlerhaften Daten verlangen kann.
- ➔ Ich weiß, dass ich jederzeit, beispielsweise beim Widerruf der Studienteilnahme, verlangen kann, dass meine bis dahin erhobenen Daten gelöscht oder unverzüglich anonymisiert werden, sodass ein Bezug zu meiner Person nicht mehr hergestellt werden kann.
- ➔ Ich erkläre, dass ich über die Erhebung und Verarbeitung meiner in dieser Studie erhobenen Daten und meine Rechte angemessen informiert wurde.
- ➔ Ich stimme der Verwendung der im Rahmen dieser Studie erhobenen Daten in der oben (oder in der Information zum Datenschutz) beschriebenen Form zu.
- ➔ Die im Rahmen der Studie erhobenen Daten können auch für künftige Forschungsvorhaben der Klinik bzw. des Instituts genutzt und weiterverarbeitet werden.
- ➔ Die Verarbeitung und Nutzung der pseudonymisierten Daten erfolgt auf Erhebungsbögen und elektronischen Datenträgern im Regelfall für die Dauer von 10 Jahren, soweit der Zweck der Studie, z. B. bei Einbringung in eine Datenbank und bei Langzeitstudien keine längere Speicherdauer erfordert.
- ➔ Die im Verlauf dieser Studie gewonnenen Informationen können für wissenschaftliche Zwecke auch an Kooperationspartner im Geltungsbereich der Europäischen Datenschutz-Grundverordnung und an Kooperationspartner außerhalb des Europäischen Wirtschaftsraumes, d.h. in Länder mit geringerem Datenschutzniveau (dies gilt auch für die USA) übermittelt werden.
- ➔ Die Studienleitung ist für die Datenverarbeitung und die Einhaltung der gesetzlichen Datenschutzbestimmungen verantwortlich.
- ➔ Für die Erhebung, Speicherung, Nutzung und Weitergabe meiner Daten ist meine ausdrückliche Zustimmung durch Unterzeichnung der Einwilligungserklärung zum Datenschutz erforderlich.
- ➔ Bei Beschwerden kann ich mich an den Datenschutzbeauftragten des Universitätsklinikums Tübingen oder den Landesdatenschutzbeauftragten des Landes Baden-Württemberg wenden.

**Einwilligungserklärung zur nachträglichen Datenerhebungen:**

- ➔ Ich erkläre, dass die Studienleitung mich nach der Beendigung der Datenerhebung erneut kontaktieren darf um bei Aufkommen neuer Forschungsfragen ergänzende Informationen zu erhalten.

**Ich erkläre, dass ich mit der im Rahmen der Studie erfolgenden Aufzeichnung von Studiendaten und ihrer Verwendung zu wissenschaftlichen Zwecken einverstanden bin.**

**Ort, Datum** **Unterschrift Proband/in**

**Ort, Datum** **Unterschrift Studienleiter/in**

**Ergänzende Informationen und Hinweise zur Änderungsmeldung vom 23.02.2021**

## 1. Informationen zur Rekrutierung

50 % der Teilnehmer der Wartekontrollgruppe aus der Hauptstudie „Randomisiert-kontrollierte Studie im Wartelisten-Kontroll-Design zur Überprüfung einer 12-wöchigen app- und orthesengestützten Trainingsintervention bei Patienten mit mittel- bis schwergradiger Gonarthrose" sollen an der Interviewbefragung teilnehmen. Davon erhielten in den letzten 12 Wochen 50% eine app-gestützte Trainingsintervention und 50% eine app-gestützte Trainingsintervention in Kombination mit dem Tragen einer Knieorthese. Per Los werden nun jeweils n=8 Teilnehmer aus beiden Subgruppen ausgewählt und befragt ob diese an einer leitfadengestützten Interviewdurchführung teilnehmen möchten. Sollten Probanden einer Teilnahme nicht zustimmen, werden weitere Probanden in entsprechender Anzahl aus den Gruppen per Los nachrekrutiert.

## 2. Informationen des Interviewer/der Interviewerin

Die Interviews werden durch zwei Mitarbeiterinnen der Abteilung Sportmedizin des Universitätsklinikum Tübingen durchgeführt, die nicht an der primären Studienplanung und Durchführung der Interventionsphase beteiligt waren. Die Studienleitung Prof. Dr. Inga Krauß sowie die in der Hauptstudie genannte Ansprechpartnerin Valerie Dieter stehen im Hintergrund für Fragen zur Verfügung.

## 3. Interviewleitfaden

Den Leitfaden mit den entsprechenden Interviewfragen finden Sie in Anlage 1.

## 4. Informationen über das Aufnahme-Medium

Je nach Probandenwunsch sollen die Interviews über Telefon oder einer Videoplattform (Zoom) durchgeführt werden. Bei einer Durchführung über Telefon wird mit einem Aufnahmegerät das Interview über die Lautsprecherfunktion aufgezeichnet. Bei Durchführung über die Videoplattform Zoom wird mittels Aufnahmefunktion des Programms das Interview aufgezeichnet.
Sollte die Ethik-Kommission Bedenken hinsichtlich einer Aufnahme der Interviews über die Videoplattform Zoom haben, so werden die Interviews ausschließlich über Telefon durchgeführt.

## 5. Umgang mit den erhobenen Audio-Daten

Interviewdurchführung über Telefon:
Alle aufgenommen Audiodaten werden pseudonymisiert gekennzeichnet und nicht an Dritte weitergegeben. Die Aufnahmen werden nach dem Interview vom Aufnahmegerät gelöscht und in einen Ordner geladen, auf den nur Studienbetreuer Zugriff haben. Die Verarbeitung und Nutzung der pseudonymisierten Daten erfolgt auf elektronischen Datenträgern im Regelfall für die Dauer von 10 Jahren, soweit der Zweck der Studie keine längere Speicherdauer erfordert.

Interviewdurchführung über die Videoplattform Zoom
Bei der Aufzeichnung über die Videoplattform Zoom wird eine Speicherung der Aufnahmedaten lokal auf dem Computer ausgewählt. Unmittelbar nach Beendigung der Aufzeichnung werden alle Dateien mit Ausnahme der Audiospur gelöscht. Mit den verbleibenden Audiodateien wird anschließend wie bei der Durchführung über Telefon umgegangen.

## 6. Weiterverarbeitung der Audio-Dateien

Im Anschluss an die Durchführung und Aufzeichnung der Interview-Audio-Dateien, werden diese transkribiert und in schriftlicher Form pseudonymisiert abgespeichert. Jegliche in der Audio-Datei vorkommenden Informationen, die eine Zuordnung zu einer bestimmten Person möglich machen, werden gelöscht/entfernt.
Auf Wunsch der Ethik-Kommission kann im Anschluss die Rohdatei der Audiospur gelöscht werden.

## 7. Auswertung der Interviews

Grundlage der Analyse ist die Erstellung eines Kategoriensystems. Als Unterstützung zur erleichterten Strukturierung und Organisation der Daten wird die Software MAXQDA verwendet.

## 8. Einverständniserklärung

Die erweiterte Einverständniserklärung mit den ergänzten Informationen finden Sie in Anlage 2. Sollte die Ethik-Kommission einer Interviewdurchführung über die Videoplattform Zoom nicht zustimmen, werden alle Interviews über Telefon durchgeführt und der entsprechende Absatz zur Interviewdurchführung über die Videoplattform Zoom aus der Einverständniserklärung der Probanden gelöscht.

**Anlage 2: Einverständniserklärung**

## Einverständniserklärung für Probanden

### Titel der Studie

**Randomisiert-kontrollierte Studie im Wartelisten-Kontroll-Design zur Überprüfung einer 12-wöchigen app- und orthesengestützten Trainingsintervention bei Patienten mit mittel- bis schwergradiger Gonarthrose**

- ➔ Bitte lesen Sie dieses Formular sorgfältig durch.
- ➔ Bitte fragen Sie den Untersucher oder Ihre Kontaktperson, wenn Sie etwas nicht verstehen oder wenn Sie mehr wissen möchten.

Sehr geehrte Teilnehmerinnen und Teilnehmer,

zum Abschluss Ihrer 12-wöchigen app-gestützten Trainingsintervention würden wir gerne ein leitfadengestütztes Interview mit Ihnen durchführen. Mittels der Interviews soll das Erleben und Bewerten der Interaktion mit der App erfasst und Hinweise zu möglichen Schwierigkeiten, Sicherheitsbedenken und Wünschen bzw. Verbesserungsvorschlägen zur Optimierung der Durchführbarkeit und Effektivität des heimbasierten, app-gestützten Trainingsprogramms eingeholt werden.

Das Interview wird leitfadengestützt mit 15 per Los zufällig ausgewählten Probanden durchgeführt. Dies bedeutet, dass der Interviewer Ihnen immer wieder Fragen stellen wird, die Sie offen und ehrlich beantworten sollen. Die Interviewdauer beträgt circa 45-60 Minuten und wird je nach Wunsch über Telefon oder die Videoplattform Zoom durchgeführt. Da das Interview für den Zweck der oben aufgeführten wissenschaftlichen Forschungsfragen ausgewertet werden soll, müssen wir das Gespräch aufzeichnen.

Alle aufgenommenen Audiodateien werden pseudonymisiert gekennzeichnet und nicht an Dritte weitergegeben. Die Verarbeitung und Nutzung der pseudonymisierten Daten erfolgt auf elektronischen Datenträgern im Regelfall für die Dauer von 10 Jahren, soweit der Zweck der Studie keine längere Speicherdauer erfordert.

Interviewdurchführung über Telefon:

Die Aufnahmen werden nach dem Interview von dem Aufnahmegerät gelöscht und in einen Ordner geladen, auf den nur Studienbetreuer Zugriff haben.

Interviewdurchführung über die Videoplattform Zoom:

Die Aufnahme erfolgt direkt über die Videoplattform Zoom. Die Speicherung der Aufnahmedaten findet lokal auf einem Computer der Abteilung Sportmedizin statt. Unmittelbar nach Beendigung der Aufzeichnung werden alle Dateien bis auf die Audiodatei gelöscht. Die Audiodatei wird in einem Ordner gespeichert, auf den nur Studienbetreuer Zugriff haben.

Das Beenden des Interviews sowie die Löschung der aufgenommenen Audio-Datei ist jeder Zeit und ohne Begründung möglich.

Im Anschluss an die Durchführung und Aufzeichnung der Interview-Audio-Dateien, werden diese verschriftlicht. Jegliche in der Audio-Datei vorkommenden Informationen, die eine Zuordnung zu einer bestimmten Person möglich machen, werden dabei gelöscht/entfernt.

Ort des Studienzentrums:

ProbandIn Vorname und Name: ______________________________

Geburtsdatum: ______________________________

Adresse/ PLZ/ Wohnort: ______________________________

______________________________

______________________________

Telefon/ E-Mail: ______________________________

______________________________

**Einwilligungserklärung zur qualitativen Befragung mittels Interviews**

- ➔ Ich stimmte zu, dass ich für ein leitfadengestütztes Interview zur Verfügung stehe und das Gespräch mit einem Aufnahmegerät / die Aufnahmefunktion der Videoplattform Zoom aufgezeichnet werden darf.
- ➔ Ich wurde darüber informiert, dass die bereits unterschriebene Einverständniserklärung bestehen bleibt und dies nur als Erweiterung zu verstehen ist.

**Ort, Datum** **Unterschrift Proband/in**

______________________________________________

**Ort, Datum** **Unterschrift Studienmitarbeiter/in**

______________________________________________
