# Supplementary material for: Patients’ experiences and usability of a self-directed m-health exercise intervention for knee osteoarthritis: a qualitative study
Source: BMJ Open. 2025 Jun 16;15(6):e100608. doi: 10.1136/bmjopen-2025-100608 (PMC12314823; doi:10.1136/bmjopen-2025-100608)
Supplement: Supplementary file 4 [file bmjopen-2025-100608.reviewer_comments.pdf]

PEER REVIEW HISTORY

BMJ Open publishes all reviews undertaken for accepted manuscripts. Reviewers are asked to complete a checklist review form and are provided with free text boxes to elaborate on their assessment. These free text comments are reproduced below.

ARTICLE DETAILS

Title (Provisional)

Patients' experiences and usability of a self-directed m-health exercise intervention for knee osteoarthritis: A qualitative study.

Authors

Dieter, Valerie; Schmidt, Joana; Krauss, Inga

VERSION 1 - REVIEW

**Reviewer**	**1**
**Name**	**Hasan, Muhammad Kamil Che**
**Affiliation**	**International Islamic University Malaysia**
**Date**	**12-Apr-2025**
**COI**	**None**

1. Expand on the potential implications of non-purposive sampling on the generalisability and transferability of findings.

2. Strengthen the reflexivity statement to clarify strategies employed to mitigate bias (e.g., reflexive journaling, independent auditing, triangulation).

3. Provide a brief statement on data saturation – was it discussed, achieved, or how was thematic sufficiency determined?

4. Consider expanding discussion on hybrid or blended care models that combine m-health with human interaction, supported by recent evidence.

**Reviewer**	**2**
**Name**	**Nielsen, Maja**
**Affiliation**	**Queen Ingrid's Hospital**
**Date**	**26-Apr-2025**
**COI**	**None**

Thank you for the opportunity to review this interesting and well-written article. I recommend a few adjustments.

Background

I would recommend defining the term *m-Health* and writing out the full expression the first time it appears in the manuscript to ensure clarity for the reader.

Please highlight the specific gaps in existing research that this study aims to address.

It would be beneficial if the authors define what is meant by "acceptability" in the context of the study. This definition would provide clarity and ensure that readers understand the specific criteria or dimensions being evaluated.

Methods

Participants section: Please explain why 14 participants were chosen and how this number was determined.

Page 9, line 39. Without addressing data saturation, it is challenging to ascertain whether the data collection process was sufficiently thorough. Please clarify why you did not discuss data saturation in your study.

Interview section: Could the authors briefly describe the key topics or questions included in the interview guide to provide more context on what was explored during the interviews?

Results

Consider providing a brief introduction to the themes identified.

Instead of writing 'The first subtheme was…', simply write the name of the subtheme, for example in italics.

Discussion

After defining 'acceptability' in the background section, make sure to use the term consistently in the discussion and address its different elements.

In general

Please add a specific section for implications for clinical practice and discuss how the findings can be applied in clinical settings. Provide specific recommendations for clinicians on how to incorporate m-health tools into patient care.

Please add a specific section for implications for future research where you suggest areas for future research. Discuss how future studies can build on the findings to further explore the effectiveness and usability of m-health interventions.

The screenshot from the app (Figure 1?) is very small. Please provide a larger version.

VERSION 1 - AUTHOR RESPONSE

Reviewer 1:

R1-1: Expand on the potential implications of non-purposive sampling on the generalisability and transferability of findings.

RESPONSE: Thank you for this comment. In the discussion section, we have already highlighted the potential risk of a selection bias resulting from non-purposive sampling. We added that this could also have implications for the generalizability of findings, as some patient groups (e.g. with less/more technical affinity, positive/negative outcomes) might be underrepresented.

Page 16, Moreover, there was a potential selection bias, as no purposive sampling (e.g. with age, sex, technical affinity, positive/negative outcomes) was included. This might have implications for the generalizability of the findings, as certain patient characteristics could be underrepresented in the study sample.

R1-2: Strengthen the reflexivity statement to clarify strategies employed to mitigate bias (e.g., reflexive journaling, independent auditing, triangulation).

RESPONSE: Thank you for this comment. Investigator triangulation was ensured by the independent development of themes and subthemes by two researchers, and the review and discussion by a third researcher. We refined the use of this approach in the discussion section. Furthermore, the utilization of audio-recording and reflexivity strategies (e.g. documentation sheets for the analysis process) in the research design served to strengthen the reliability of the findings. A sentence was added to reflect this statement. An independent audit was not conducted.

Page 15, Using an investigator triangulation approach, themes and subthemes were independently developed by two researchers (JS, VD), and subsequently reviewed and discussed by a third researcher (IK).

Page 15, Furthermore, the utilization of audio-recording, the aforementioned triangulation, and reflexivity strategies (e.g. documentation sheets for the analysis process) in the research design served to strengthen the reliability of the findings.

R1-3: Provide a brief statement on data saturation – was it discussed, achieved, or how was thematic sufficiency determined?

RESPONSE: Thank you for this request. As described in the methods subsection “Interviews” (see page 7), data saturation was not discussed. In regard to the sample size, the present study was aligned with other qualitative research studies that examined digital exercise delivery for patients with knee osteoarthritis. In doing so, it was hypothesized that a sufficient amount of data would be collected to achieve data saturation. Thematic saturation was ensured by using a reflective analysis process, with forward and backward movement within the different steps (see page 7-8, data analysis section), and by using a systematic and transparent process of developing the category system, coding, and generation of themes and subthemes (see pages 7-8, data analysis section). The two researchers (JS, VD), who were developing themes and subthemes, independently perceived that no new thematic content was arising when coding the last 4 interviews.

Page 5, Participants

[…]. The sample size was specified a priori according to previous qualitative research studies examining digital exercise interventions for patients with knee OA [11, 12].

R1-4: Consider expanding discussion on hybrid or blended care models that combine m-health with human interaction, supported by recent evidence.

RESPONSE: Thank you for this request. We expanded the discussion on hybrid or blended care models that combine digital exercise delivery with human interaction by incorporating further studies using telehealth-only or combined telehealth and in-person care for exercise delivery. In this regard, the term "hybrid" has been restricted not only to blended care models, but also extended to digital delivery tools using synchronous digital human interaction (e.g. telehealth). Thereby, the potential of adequately preparing patients for upcoming exercise sessions (e.g. by empowering them for correct and safe exercise technique, answering questions, providing reassurance, or managing technological issues) when incorporating human interaction was discussed.

Page 15, […] The ability of physical therapists to tailor exercise programs according to patients' needs was described as a key benefit in a blended care intervention [27]. […]. In addition, the incorporation of human interaction with a health professional via telehealth or blended care in digital exercise delivery may address the importance of adequately preparing patients for upcoming digital exercise sessions by empowering them for correct and safe exercise technique, answering questions, providing reassurance, or managing technological issues [14, 30].

Reviewer 2:

Background

R2-1: I would recommend defining the term *m-Health* and writing out the full expression the first time it appears in the manuscript to ensure clarity for the reader.

RESPONSE: Thank you for this request. We have included a definition of the umbrella term digital health and introduced the categories e-health, m-health, and telehealth.

Page 3, In order to address the current deficiencies in healthcare, the utilization of digital health technologies in the delivery of exercise programs could be increased in the future. It can be assumed that this could additionally facilitate patients' access and adherence to unsupervised home-based exercise. By definition, digital health technologies integrate information and communication tools that can be utilized to improve accessibility and efficiency of healthcare [8, 9]. In this context, digital health is an umbrella term that encompasses electronic health (e-health), mobile health (m-health), and telehealth [9], whereby m-health technologies specifically focus on mobile communication tools [10]. Overall, […].

R2-2: Please highlight the specific gaps in existing research that this study aims to address.

RESPONSE: Thank you for this comment. This aspect has been considered and integrated.

Page 4, […] To date, most qualitative studies on patients with knee OA have investigated the feasibility and acceptability of web-based programs [11, 12] or supervised digital delivery modes (e.g. Skype) [14]. Consequently, there remains a limited understanding of patients' experiences, usability, and

acceptability when utilizing a self-directed app-delivered and sensor-assisted exercise intervention for patients with knee OA. Ultimately, addressing this gap is crucial for the successful implementation of a program [15]. In this context, existing studies on digital exercise programs have largely overlooked the distinction between different types of digital health technologies. However, this is a critical oversight, as varying levels of technological support may present unique challenges for patients, potentially influencing their engagement and outcomes.

R2-3: It would be beneficial if the authors define what is meant by "acceptability" in the context of the study. This definition would provide clarity and ensure that readers understand the specific criteria or dimensions being evaluated.

RESPONSE: Thank you for this suggestion. The term "acceptability" was defined as a construct that reflects the evaluation of the m-health exercise app as a suitable method for delivering an exercise intervention, based on patients' experiences and needs.

Page 4, […]. This qualitative examination aimed to identify patient's perspectives on facilitators and barriers, usability, and acceptability of a self-directed app- and sensor-delivered exercise intervention. In this context, "acceptability" was defined as a construct that reflects the evaluation of the m-health exercise app as a suitable method for delivering an exercise intervention, based on patients' experiences and needs.

Methods

R2-4: Participants section: Please explain why 14 participants were chosen and how this number was determined.

RESPONSE: Thank you for this comment. The sample size was specified a priori according to previous qualitative research studies examining digital exercise interventions for patients with knee osteoarthritis. We added this within the participants section. In addition, we added a statement within the limitation section.

Page 5, The sample size was specified a priori according to previous qualitative research studies examining digital exercise interventions for patients with knee OA [11, 12].

Page 16, Furthermore, the sample size was determined a priori. However, despite this limitation, inductive thematic saturation, as well as a priori thematic saturation could be confirmed in the data set [30].

R2-5: Page 9, line 39. Without addressing data saturation, it is challenging to ascertain whether the data collection process was sufficiently thorough. Please clarify why you did not discuss data saturation in your study.

RESPONSE: As mentioned above, the sample size of this study was specified a priori according to previous qualitative research studies that examined digital exercise interventions for patients with knee osteoarthritis. In doing so, it was hypothesized that a sufficient amount of data would be collected to achieve data saturation. In addition, thematic saturation was ensured by using an iterative and reflective analysis process, with forward and backward movement within the different steps (see page 7-8, data analysis section), and by using a systematic and transparent process of developing the

category system, coding, and generation of themes and subthemes (see page 7-8, data analysis section). The two researchers (JS, VD), who were developing themes and subthemes, independently perceived that no new thematic content was arising when coding the last 4 interviews. We summarized this procedure concisely with the following sentence which was added to the limitation section of the manuscript: “Furthermore, the sample size was determined a priori. However, despite this limitation, inductive thematic saturation, as well as a priori thematic saturation could be confirmed in the data set [30].”

Page 16, Furthermore, the sample size was determined a priori. However, despite this limitation, inductive thematic saturation, as well as a priori thematic saturation could be confirmed in the data set [30].

R2-6: Could the authors briefly describe the key topics or questions included in the interview guide to provide more context on what was explored during the interviews?

RESPONSE: Thank you for this suggestion. We added a sentence including the key topics.

Page 7, The interview guide was developed by the authors VD (M. Sc. Sports Science, PhD candidate, RCT coordinator) and IK (apl. Prof., sports scientist and physiotherapist) and addressed patients' (positive and negative) experiences with the app and its features during the course of the exercise intervention. In addition, the patients’ needs and perspectives on usability, the design of the app, and suggestions for improvement to optimize app functionality were gathered.

<u>Results</u>

R2-7: Consider providing a brief introduction to the themes identified. Instead of writing 'The first subtheme was…', simply write the name of the subtheme, for example in italics.

RESPONSE: Thank you for this suggestion. We have replaced the opening sentence of the subthemes “The first subtheme was ...” with a subheading written in italics referring to the name of the subtheme (e.g. *Flexibility*).

Page 9, *App features for guidance, support and monitoring of correct exercise execution*

[…]

Page 10, *Flexibility*

[…]

Page 10, *App features to increase motivation and regularity of the exercise program*

[…]

Page 11, Positive experiences with the exercise program

[…]

Page 11, *No on-site instruction by a health professional*

[…]

Page 11, *Technical issues*

[…]

Page 12, *Difficulties with the exercise program*

[…]

Page 12, *Associated problems with the app features*

[…]

Page 12, *Usability, feasibility and comprehensibility*

[…]

Page 12, *Handling and screen size*

[…]

Page 13, *Positive outcomes*

[…]

Page 13, *Not met outcome expectations*

[…]

Discussion

R2-8: After defining ‘acceptability’ in the background section, make sure to use the term consistently in the discussion and address its different elements.

RESPONSE: The term "acceptability" should now have been employed consistently throughout the discussion section, thereby addressing the elements in relation to patients' experiences and needs when utilizing the m-health exercise intervention.

In general

R2-8: Please add a specific section for implications for clinical practice and discuss how the findings can be applied in clinical settings. Provide specific recommendations for clinicians on how to incorporate m-health tools into patient care.

RESPONSE: Thank you for this comment. We changed the title from “Conclusion” to “Conclusion and implication for clinical practice”. In addition, a statement regarding implications for clinical practice has been incorporated into this section.

Page 16, Conclusion and implications for clinical practice

[…]. For clinical practice, this study demonstrates that self-directed digital exercise interventions are not a “one-size-fits-all” solution. Instead, potential advantages and disadvantages should be discussed with the individual patient, as personal and environmental context factors (e.g. level of support,

previous experience in physical exercise, technical affinity, access to health care providers) need to be considered when selecting the appropriate treatment option. […].

R2-9: Please add a specific section for implications for future research where you suggest areas for future research. Discuss how future studies can build on the findings to further explore the effectiveness and usability of m-health interventions.

RESPONSE: Thank you for this suggestion. We added the implications for future research at the end of the conclusion and implications for clinical practice section.

Page 15, […] Moreover, future research should focus on the comparison between supervised and self-directed digital exercise delivery in knee OA. In this context, it is crucial to identify the responder characteristics for the different delivery modes, thus enabling the recommendation of a best-fit solution for each patient, tailored to their individual characteristics.

R2-10: The screenshot from the app (Figure 1?) is very small. Please provide a larger version.

RESPONSE: We will upload a larger version of the screenshot.

Editor's Comments to Author:

E1: Please can you move the text description of the participants' characteristics and table 1 from the methods section to the beginning of the results section?

RESPONSE: Thank you for this suggestion. The text description of the participants' characteristics and table 1 were moved from the methods section to the beginning of the results section. A new subheading "Participant characteristics" was added.

Page 9, Results

Participant characteristics

Out of 16 randomly selected participants, 14 gave consent were interviewed between May 06, 2021 and May 19, 2021. All participants completed the interview within a time window of 2-5 weeks after intervention completion. Two other participants declined to participate. One out of time reasons in combination with a rehabilitation stay and one due to lack of interest. The average duration of the interviews was 57 minutes. Table 1 reports the baseline characteristics of the interviewed participants.

*Table 1

VERSION 2 - REVIEW

**Reviewer**	**2**
**Name**	**Nielsen, Maja**
**Affiliation**	**Queen Ingrid's Hospital**
**Date**	**21-May-2025**

COI

Thank you for the opportunity to review the revised manuscript. The authors have thoroughly addressed all of my comments and suggestions, and the revisions have clearly improved the quality and clarity of the paper. I have no further comments and recommend the manuscript for acceptance in its current form.
